# Substrate complexity and species number jointly shift the balance between competition and facilitation in a chitin-degrading bacterial consortium

**DOI:** 10.1093/ismeco/ycag272

**Published:** 2026-09-25

**Authors:** Moritz Mohrlok, Lauren Alteio, Julia Berger, Ksenia Guseva, Erika Salas, Monika Schmoll, Dave Sirbu, Christina Kaiser

**Affiliations:** Department for Microbiology and Ecosystem Science, Division of Terrestrial Ecosystem Research, University of Vienna, Centre for Microbiology and Environmental Systems Science, A-1030, Vienna, Austria; University of Vienna, Doctoral School in Microbiology and Environmental Science, A-1030, Vienna, Austria; Department for Microbiology and Ecosystem Science, Division of Terrestrial Ecosystem Research, University of Vienna, Centre for Microbiology and Environmental Systems Science, A-1030, Vienna, Austria; Austrian Competence Centre for Feed and Food Quality, Safety and Innovation, FFoQSI GmbH, A-3430, Tulln, Austria; Department for Microbiology and Ecosystem Science, Division of Terrestrial Ecosystem Research, University of Vienna, Centre for Microbiology and Environmental Systems Science, A-1030, Vienna, Austria; Department for Microbiology and Ecosystem Science, Division of Terrestrial Ecosystem Research, University of Vienna, Centre for Microbiology and Environmental Systems Science, A-1030, Vienna, Austria; Department for Microbiology and Ecosystem Science, Division of Terrestrial Ecosystem Research, University of Vienna, Centre for Microbiology and Environmental Systems Science, A-1030, Vienna, Austria; Department for Microbiology and Ecosystem Science, Division of Terrestrial Ecosystem Research, University of Vienna, Centre for Microbiology and Environmental Systems Science, A-1030, Vienna, Austria; Department for Microbiology and Ecosystem Science, Division of Terrestrial Ecosystem Research, University of Vienna, Centre for Microbiology and Environmental Systems Science, A-1030, Vienna, Austria; Department for Microbiology and Ecosystem Science, Division of Terrestrial Ecosystem Research, University of Vienna, Centre for Microbiology and Environmental Systems Science, A-1030, Vienna, Austria

**Keywords:** microbial interactions, soil bacteria, synthetic community, substrate complexity, chitin degradation, cooperation, competition, consumer-resource modeling

## Abstract

Biopolymer-degrading microbial communities exhibit complex interactions like division of labor, sharing of public goods, and resource-competition, resulting in intricate social dynamics between their diverse members that shape the functions they perform. How these interactions shift with substrate complexity as species number increases remains largely unresolved, especially as substrate type can fluctuate. Here, we investigated how these factors modulate microbial interactions and community functioning in a synthetic three-species chitin-degrading soil bacterial consortium. All mono-, duo-, and tri-cultures were grown under well-mixed conditions on a labile substrate (N-acetylglucosamine, NAG), a complex polymer (chitin), or a combination of both. We quantified respiration, final biomass, and community structure, chitinase activities, degradation products and cross-feeding responses, and integrated these data with consumer-resource modeling. Substrate complexity altered interaction patterns. On NAG, we frequently observed competitive exclusion; while on chitin less competitive species were still detectable at the end. This suggests a shift from competition toward facilitation. The tri-culture showed significant final biomass increases on NAG, likely related to cross-feeding effects and enhanced polymer degradation and respiration on chitin, indicating multi-species facilitation. Model-experiment discrepancies indicate that competition for primary resources and decomposition product-sharing alone cannot explain the observed community dynamics, suggesting additional metabolic and behavioral interactions. While it remains to be tested whether our findings translate beyond the well-mixed conditions studied here, they show how the balance between positive and negative interactions in this consortium shifts with substrate complexity and species number, shaping community structure and functioning.

## Introduction

The composition and functions of microbial communities emerge from a network of interactions among their members [1]. Positive interactions like waste removal, cross-feeding or division of labor, can enhance community diversity, productivity and efficiency [2–5]. In contrast, negative interactions, like competition or predation, can lead to species extinction [6] but also promote community stability by dampening positive feedback loops [7, 8]. As positive and negative interactions have opposing effects on community-level properties, understanding the conditions that favor one interaction type over the other is crucial for our understanding of community function.

While there is abundant evidence for the dominance of negative interactions across various systems, from soil to gut [8], recent work suggests that positive interactions among bacteria might be more frequent than previously thought [1]. However, most research on bacterial interactions relies on co-culturing taxa using simple organic substrates or standard culture media, neglecting the potential effect of substrate complexity on interactions. This is, however, essential, as many natural microbial communities thrive by degrading widespread complex biopolymers from natural sources like chitin, cellulose, or lignin as well as environmental pollutants [9].

Degradation of such polymers requires the production of various extracellular enzymes. Chitin for example, nature’s second most abundant biopolymer [10], is mainly degraded by synergistic exo- and endochitinases targeting different sites of its N-acetylglucosamine (NAG) chains [11]. See supplementary material section S2.1 for a more detailed overview of chitin degradation mechanisms. As producing different enzymes is costly for individual cells, this frequently leads to division of labor and resource-sharing, increasing overall community efficiency [2]. Such communities often exhibit cross-feeding on secreted metabolic byproducts, which removes toxins [12], increases overall carbon-use efficiency [3] and diversifies the community [13]. Conversely, as decomposition products are available to all nearby microbes, exploitation of degraders by non-enzyme producing microbes can diminish decomposer efficiency [10, 14]. Therefore, these systems exhibit intricate interactions and cascading effects, leading to emergence and succession of various functional groups [10, 13, 15, 16]. Degraders produce extracellular enzymes, exploiters take up their products without participating in degradation and scavengers only utilize metabolic byproducts instead of the main substrates [10]. This diversity of roles and interactions among them drives complex behavior, in contrast to simple competition for readily available substrates such as sugars. Despite the important role of microbial biopolymer degradation in nature, the underlying effects that substrate complexity can have on interaction patterns in degrader communities remain sparingly understood.

Studies indeed show that substrate complexity fosters positive interactions and synergistic growth among community members, whereas simple substrates promote negative interactions and competition [17–21]. Most work in the field, with few exceptions, has focused on pairwise communities [18, 20, 21], providing a limited view of the studied systems, as multi-species interactions are important to the understanding of diverse communities [22, 23], and might already be predicted by tri-cultures [24]. Although those have been included in some studies [17, 19], a coherent understanding of how interaction patterns and community functioning shift with different substrate conditions and species numbers remains elusive.

The combined effects of complex and labile substrates have to our knowledge also not yet been investigated in co-culturing experiments. This is relevant, as the complexity of substrates to which natural decomposer communities are exposed, fluctuates frequently. For instance, soil bacterial communities in the vicinity of roots that primarily degrade complex organic compounds irregularly experience additional input of simple substrates by pulses of root exudation [25, 26]. Labile substrates may also become more available in soils during the growing season of plants [20]. More systematic evidence on how interactions in bacterial communities can change across labile and complex substrate regimes and what effect this has on community functions is needed, especially as labile C input dynamics into various ecosystems are affected by global change [27–30].

To address this, we studied a synthetically assembled decomposer consortium consisting of three not commonly studied soil bacteria that can grow in pure culture under similar incubation conditions and have the genomic potential to degrade chitin and utilize its easily assimilable monomer NAG: (i) *Paenibacillus alginolyticus*—PA, derived from a rhizosphere-associated genus known for biopolymer degradation and plant-growth promotion [31]; (ii) *Paraburkholderia xenovorans*—PX, isolated from contaminated soil and a model aromatic-compound degrader [32]; and (iii) *Solirubrobacter soli*—SS, a slow-growing actinobacterium isolated from ginseng-field soil [33]. We combined them in all possible combinations (mono-, duo-, and tri-culture) on either the labile (NAG) or the complex (chitin) substrate, or a combination of both (NAG+chitin), and measured cumulative respiration and final cell abundances as indicators of community activity and productivity. We incorporated functional measurements of enzyme activities, chitin breakdown products and cross-feeding to gain mechanistic insights into system functioning. Using a mathematical consumer-resource model, we predicted community respiration in duo- and tri-cultures based on mono-culture parameterization and baseline resource-driven interactions. We then compared these predictions to experimental data to identify shifts in respiration that could not be explained solely by competition for and sharing of primary resources.

From these functional and mechanistic data, we inferred the interactions present in each combination and assessed how the balance between competition and facilitation changes with substrate complexity and species number. Based on known social dynamics in degrader communities, we hypothesized that the presence of chitin fosters positive interactions, while increasing the abundance of NAG leads to competitive exclusion. We also hypothesized that community-level outcomes in three-species consortia are not fully predictable from pairwise interactions alone.

## Materials and methods

### Bacterial strains

To obtain a potentially chitin-degrading consortium, we screened a collection of soil bacterial strains for potential NAG metabolism and hydrolytic chitinase production using the MetaCyc database [34] and Pathway Tools [35], see supplementary material section S2.2. All chosen strains—PA DSM 5050/1347, PX DSM 17367/LB400, and SS DSM 22325/Gsoil 355—had the genomic potential to utilize NAG. Strains were obtained from the German Collection of Microorganisms and Cell Cultures GmbH (DSMZ). The bacteria were maintained in their respective optimal medium (DSMZ M1 for PA, LB for PX, and R2A for SS) at 28°C before the experiments. Their genomes were additionally screened for carbohydrate-active enzyme encoding genes to identify unknown enzymes potentially involved in chitin degradation (S3).

### Cumulative respiration

Cumulative respiration as a proxy for community activity was measured using the MicroResp^TM^ system. Three cultures of each species were harvested, washed, and diluted to similar cell concentrations, checked with a cell counter (Quantom Tx, Logos Biosystems). Duo- and tri-cultures were prepared by mixing mono-cultures in equal volumetric ratios (50:50 or 33:33:33), keeping total cell numbers approximately constant (S5). MicroResp setups contained three treatments with increasing ratios of complex to labile substrate: M9 minimal medium [36] + NAG (2% w/v), M9 + colloidal chitin (2%), M9 + colloidal chitin + NAG (1% + 1%), and one treatment containing M9 only. Inoculated setups were incubated at 28°C, shaking at 100 rpm. CO_2_ evolution was assessed by measuring and changing the MicroResp absorbance plates daily for 2 weeks. CO_2_ concentration in the headspace was calculated from indicator absorbance shift at 540 nm and converted to μg CO_2_-C per time interval. Each timepoint was corrected by the background respiration of the respective community incubated with M9 medium. After the experiment, technical replicates were pooled to obtain sufficient volume for subsequent measurements. A detailed method description, including for the following sections, is provided in the supplement (S8).

### Final cell counts and community composition

As the MicroResp setups and particulate chitin prohibited time-resolved biomass measurements based on turbidity, we assessed final community composition and gene copy numbers in the pooled samples via 16S ribosomal ribonucleic acid (rRNA) amplicon sequencing and digital-droplet polymerase chain reaction (ddPCR). While this method of quantification can be biased by multiple factors (variations in 16S gene copy numbers, primer affinities, extraction efficiency), we assume any introduced bias to be consistent across samples. We also refer to the derived copy numbers as “biomass” from here on for simplicity, acknowledging that we do not capture changes in cell mass or carbon content with this approach. Deoxyribonucleic acid (DNA) was extracted by heating resuspended pellets to 95°C in a thermal cycler (T100, BioRad Laboratories) for 10 minutes [37]. The V4 region of the 16S rRNA genes was amplified following a two-step PCR barcoding approach using primers 515F and 806R [38]. Amplicons were sequenced on a MiSeq platform (Illumina, V3 chemistry, 600 cycles) at the Joint Microbiome Facility (JMF, Vienna, Austria). Reads were converted to relative abundances using the R-package “phyloseq” [39]. To quantify 16S rRNA genes, ddPCR was performed [40], using the same primers. Absolute abundance of each species was calculated by multiplying total 16S gene copy number with species’ relative abundance and dividing by average 16S genes in its genome (NCBI: 4 for PA, 9 for PX, 3 for SS*)* to obtain a proxy for cell numbers [41]. Cross-contaminated samples containing target genera (*Paenibacillus*, *Paraburkholderia*, *Solirubrobacter*) where they should not be present based on treatment or samples containing only non-target genera were removed from analysis (15 in total). As short-read sequencing provides low species-level resolution [42], we summed up the relative abundance of amplicon sequence variants (ASVs) with our target genera but lacking a species name, confident that they represent the introduced species and not contaminations. We also confirmed that these sequences had a higher than 99% similarity to the target.

### Chitin decomposition products and chitinase activity

Final chitooligomer concentrations were measured in the sample supernatants using an ultra-performance liquid chromatography and Orbitrap mass-spectrometry approach combined with pre-column 1-phenyl-3-methyl-5-pyrazolone (PMP)-derivatization [43], which allows sensitive detection and quantification of NAG oligomers. Chitinase activity was measured in a separate microplate experiment using identical substrate and incubation conditions as before. After ten days of incubation, current N-acetylglucosaminidase, exo-, and endochitinase activities were assayed using 4-methylumbelliferone (MUF)-labeled NAG, chitobiose, and chitotriose, respectively.

### Cell-free spent media experiment

Possible metabolic interactions between the three species were tested in a cell-free spent media (CFSM) experiment. Mono-cultures were grown in the same media as in the MicroResp experiment for ten days and then filtered to obtain cell-free supernatants, further used as media for new cultures, alongside fresh and uninoculated medium as controls. Each species was grown in its own and the spent media of all others across all substrates under identical conditions in microplates. Growth (OD_600_) was assessed daily for 10 days, using the maximally reached OD_600_ as a measure of overall performance.

### Statistical analysis

Nonlinear least-squares (NLS) regressions were fitted to the cumulative respiration data for each strain/substrate combination across all biological replicates and tested against a null model fit to all data within a substrate/species number subgroup, multiple testing was handled using Benjamini–Hochberg correction. Non time-resolved data was assessed with two-way analyses of variance (ANOVAs) testing the effect of species combination and substrate (for total cumulative CO_2_, total final cell numbers and the abundance of individual species) or one-way ANOVAs testing the effect of species combination within each substrate (chitooligomer concentration, chitinase activity, maximum OD_600_ in CFSM). Statistical tests were done on biological replicate level, calculating the mean of technical replicates beforehand. ANOVAs were followed by Tukey’s HSD post-hoc test. If ANOVA assumptions were violated, a nonparametric Kruskal–Wallis test followed by Dunn’s post-hoc test with Benjamini–Hochberg correction was performed instead.

### Consumer-resource modeling

To identify interactions that go beyond competition for primary resources and sharing of enzymatic products, we compared our results with the outcome of a consumer-resource model, which only includes primary resource-based interactions (S6.2b). It consists of different carbon (C) pools coupled by ordinary differential equations and simulates bacteria degrading chitin, taking up NAG, growing, dying and respiring. It assumes: (i) NAG is the only C source; (ii) all species compete for NAG; and (iii) the products of activity of extracellular enzymes produced by PA are available to all species. To incorporate known density-dependent effects on microbial growth [44, 45], the model further assumes that (iv) growth is limited by an additional factor depending on maximal biomass. Based on these assumptions, the model only includes basic interactions: competition for NAG and sharing of public goods (enzymes) produced by PA (S6.2). More complex resource-based interactions like the sharing of metabolic by-products are not implemented in this simple model. A detailed explanation on initial conditions, assumptions, and dynamics is provided in the supplement (S6). The model was parametrized on the measured mono-culture respiration using a Markov-Chain Monte Carlo approach [46] and simulations were run over the experimental timespan for all treatments. Initial conditions were estimated from the C concentrations of the experiment. We interpreted deviations between measured and simulated respiration in duo- and tri-cultures as indicators for processes not included in the model.

## Results

### Substrate complexity and species combinations drive cumulative respiration

Microbial activity was approximated by measuring cumulative respiration over time. Both substrate and species combination significantly affected the total respired C (*P* < .001, S1). Respiration was generally reduced by an order of magnitude in chitin compared to NAG-containing treatments (Fig. 1). In almost all cases (except PA + PX duo-culture and SS mono-culture on chitin), respiration peaked within 100–200 hours for most treatments and declined after that (S4), resulting in a flattening of the cumulative curves (Fig. 1).

**Figure 1:**
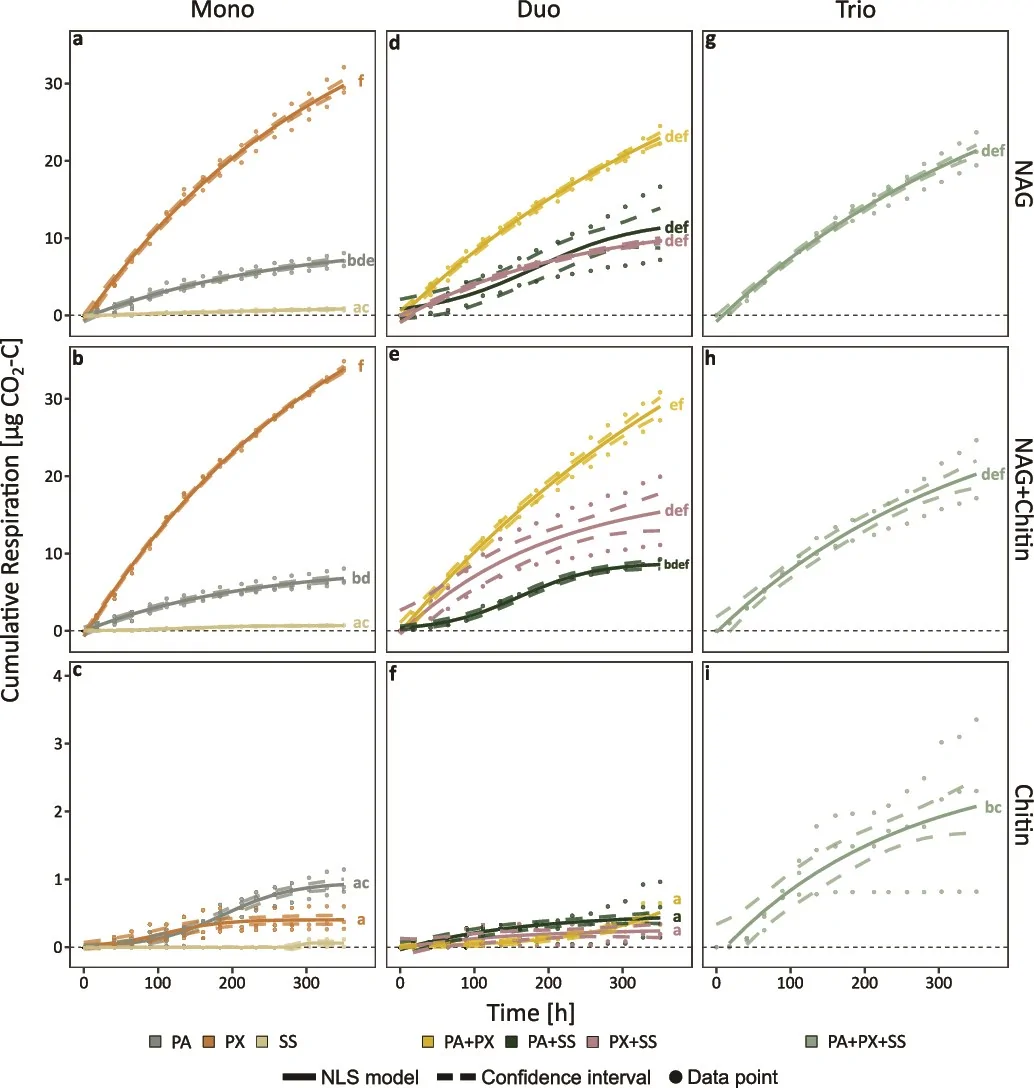
Cumulative respiration. Species combinations, substrate complexity and their interactions affect total C respiration. Panels show cumulative CO_2_ production over time by mono-, duo- and tri-cultures of a chitin-degrading bacterial consortium incubated with substrates of varying complexity. Dots show cumulative respiration of 3 biological replicates (each being an average of 3 technical replicates), measured at 15 timepoints, corrected for the background-respiration in carbon-free M9 minimal media. Lines represent nonlinear regression models and their 95% confidence intervals (dashed) that were fit to the measured data by pooling biological replicates (one model per treatment, e.g. PA-NAG). To test differences between species and their combinations, each model was compared to a null model fitted to all data within its biodiversity (mono, duo, trio) treatment and substrate combination (e.g. NAG-mono) with an F-test (see S1.2). For the tri-culture, this test was performed across all three substrates. Except for PX + SS-chitin, all models were significantly different from the respective null model (S1.2), showing that species metabolic repertoire and pairwise combinations influenced respiration rates in the mono- and duo-cultures, while substrate complexity influenced respiration rates in the tri-culture. Total respirated carbon at the incubation endpoint was compared across all substrate and species combinations, and both factors and their interaction were found to have significant effects (two-way ANOVA, *P* < .001 for all factors, see S1.1). Lowercase letters next to curves indicate significant differences in final respiration between treatments obtained from a TukeyHSD post-hoc test. F = F-value, *P* = *P*-value.

The three mono-cultures exhibited distinct responses to the substrates, as demonstrated by the significant differences in their cumulative respiration compared to the respective NLS null model, which is based on all mono-cultures for a given substrate (Fig. 1a–c, S1.2). PX respired the most in NAG and NAG+chitin, followed by PA (Fig. 1a and b). Similar respiration of the two species in NAG and NAG+chitin with NAG still being present at the end (S7) suggests that growth was not limited by NAG, but by some unassessed factor. In chitin, PA showed the highest cumulative respiration, followed by PX (Fig. 1c). SS exhibited minimal respiration compared to the others, regardless of substrate.

Respiration patterns in the combinations changed significantly depending on species and substrate treatment. Respiration curves of PX-containing cultures in NAG and NAG+chitin had a similar shape as its mono-cultures, but overall levels were lower (Fig. 1d, e, g, and  h). PA + SS reached similar or even higher levels of respiration compared to their mono-cultures in the NAG-containing treatments (Fig. 1d and e).

Respiration on chitin was generally lower in the duo-cultures than in the mono-cultures (Fig. 1c and f). The tri-culture exhibited significantly higher respiration than all duo-cultures (Fig. 1f and i) and similar or higher levels than the mono-cultures that ramped up earlier (Fig. 1c and i).

### Chitin increased the endpoint abundance of less competitive species

Cell abundances in the final samples were assessed to evaluate community composition across treatments. Both substrate complexity and species combinations significantly affected the final biomass of individual species and the total community (Fig. 2, S1.3). In the NAG-treatments, mono-culture cell numbers mirrored cumulative respiration patterns (Fig. 1a and b): PX reached the highest final abundance, followed by PA, while SS had the lowest values (Fig. 2a and d). Similarly, the abundance of PX on chitin was significantly lower (Fig. 2g). Despite respiration differences, PA showed similar final abundances across all substrates, while SS exhibited low cell numbers in the NAG treatments but similar values to the other species on chitin (Fig. 2a, d, and  g).

**Figure 2:**
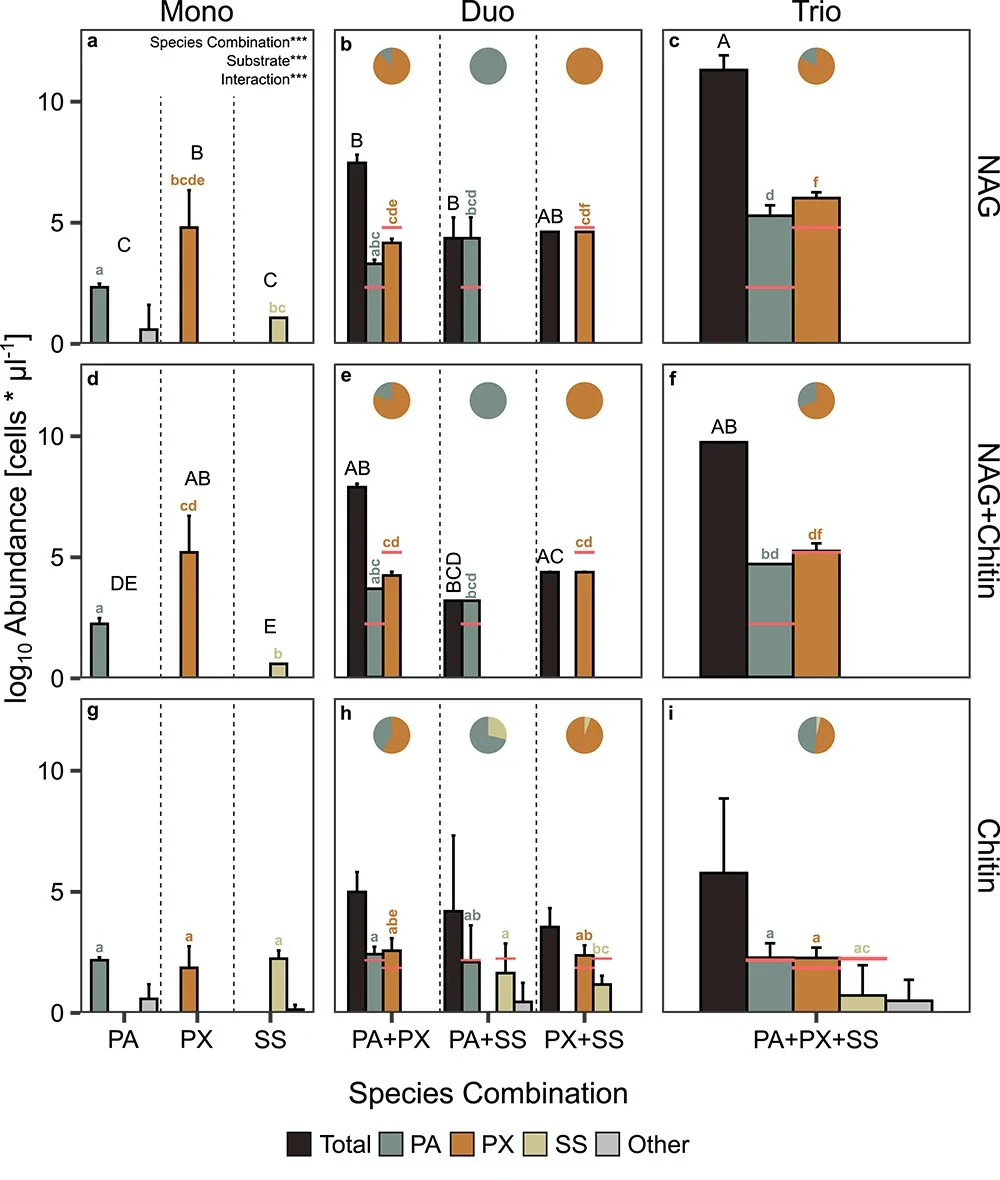
Final community composition. Substrate complexity and species combination affect individual species and total community biomass. Bars show a proxy of absolute abundances of each species in the final communities obtained from 16S rRNA amplicon sequencing and ddPCR, corrected by genome copy number, after 2 weeks of incubation in the MicroResp system (mean ± standard deviation, *n* = 3), as well as total community abundance. Horizontal lines on bars of individual species in duo-and tri-culture correspond to the mean abundance of the respective species in its monoculture. Pie charts represent the relative abundances of each species. Species combination, substrate and their interaction had significant effects on the final abundance of each individual species and the total community (two-way ANOVA, S1.3). Uppercase letters indicate the results of a Tukey-HSD post-hoc test comparing the effect of species combinations on total community biomass within each substrate (i.e. can be only compared within one row of panels). Lowercase letters indicate significant differences in absolute abundance of target species across all substrates and combinations and can only be compared within one species.

The highest total abundances were found in the tri-culture growing on NAG, followed by NAG+chitin (Fig. 2c and f), indicating potential growth facilitation not reflected in the respiration curves which were lower than the highest mono-cultures. PX dominated, particularly when NAG was present (Fig. 2b and e), in line with the respiration measurements. In NAG-containing treatments, PA showed increased abundance compared to its mono-culture when combined with PX or SS (showing a significant trend). Although SS was not detected at the final timepoint, this suggests a transient interaction also reflected in the respiration data in which SS initially promoted the growth of PA.

The endpoint relative abundance of less competitive species in NAG-containing treatments, such as SS or PA in combination with PX, was increased when chitin was present. The relative proportion of PA to PX increased with a decreasing proportion of initial labile substrate (Fig. 2b, e, and  h). Similarly, SS was only detectable after the experiment in the chitin-only treatments, although only in one pooled technical replicate in the tri-culture (the one with the highest respiration, Fig. 1i). While not statistically significant, the tri-culture additionally exhibited the highest final total cell number of all chitin-only treatments (Fig. 2g–i), on average more than ten times higher than the duo-cultures (log_10_ fold change of 1.15 ± 0.97).

### The species showed different chitin decomposition potential

Next, to determine whether differences in chitin degradation potential also translated into functional differences, we measured chitooligomer concentrations and chitinase activity. Significant chitinase activity and oligomer concentrations were only measurable when PA was present (Fig. 3). This indicates that PA was the only species degrading chitin in our experiment, despite the genomic potential of the others (S2.2), in line with the genome screening result (S3). Exo- and endochitinase activity was highest in the PA + SS combination, although not significantly different from the PA monoculture and the tri-culture.

**Figure 3:**
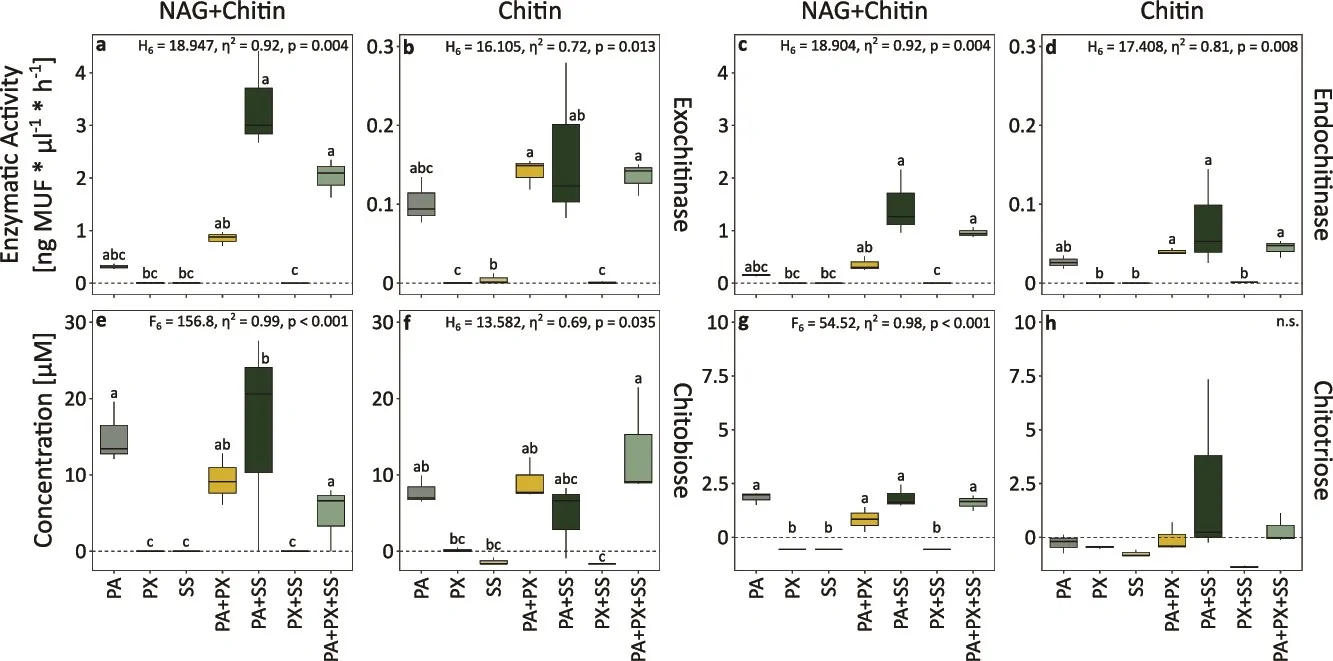
Chitin decomposition. Significant chitinase activities and chitin breakdown products are only measurable when PA is present. Boxplots (median and interquartile range, *n* = 3) show exochitinase (a, b) and endochitinase (c, d) activity in the supernatant of mono-, duo- and tri-cultures of a soil bacterial decomposer consortium after 10 days of incubation with NAG+chitin or chitin. Subplots e–h show the blank-corrected measured concentration of the chitin oligomers chitobiose (e, f) and chitotriose (g, h) in the supernatant of pooled technical replicates (*n* = 3 each, three biological replicates) at the end of a 2-week incubation experiment (see Fig. 1). Negative values are a result of native oligomer concentrations in the blanks of chitin-containing media. Test results correspond to an ANOVA, H-values to a Kruskal–Wallis test when ANOVA assumptions were not met. Lowercase letters indicate significant differences between groups (within each panel).

### Putative metabolic interactions changed with substrate

To explore how metabolites produced by one species under specific substrate conditions influence the growth of other species, we measured the maximum biomass of species grown in each other’s and their own byproducts (spent medium from previous incubation with same substrates), interpreting differences as potential signals of metabolic interactions. We also compared growth in spent media with growth of the respective strains in filtered fresh substrate (Fig. 4) to assess how different levels of essential nutrient depletion potentially limit growth, obscuring interaction signals. Note that the spent media did not contain any chitin particles, but only accumulated metabolites, leftover NAG or dissolved chitooligomers produced beforehand.

**Figure 4:**
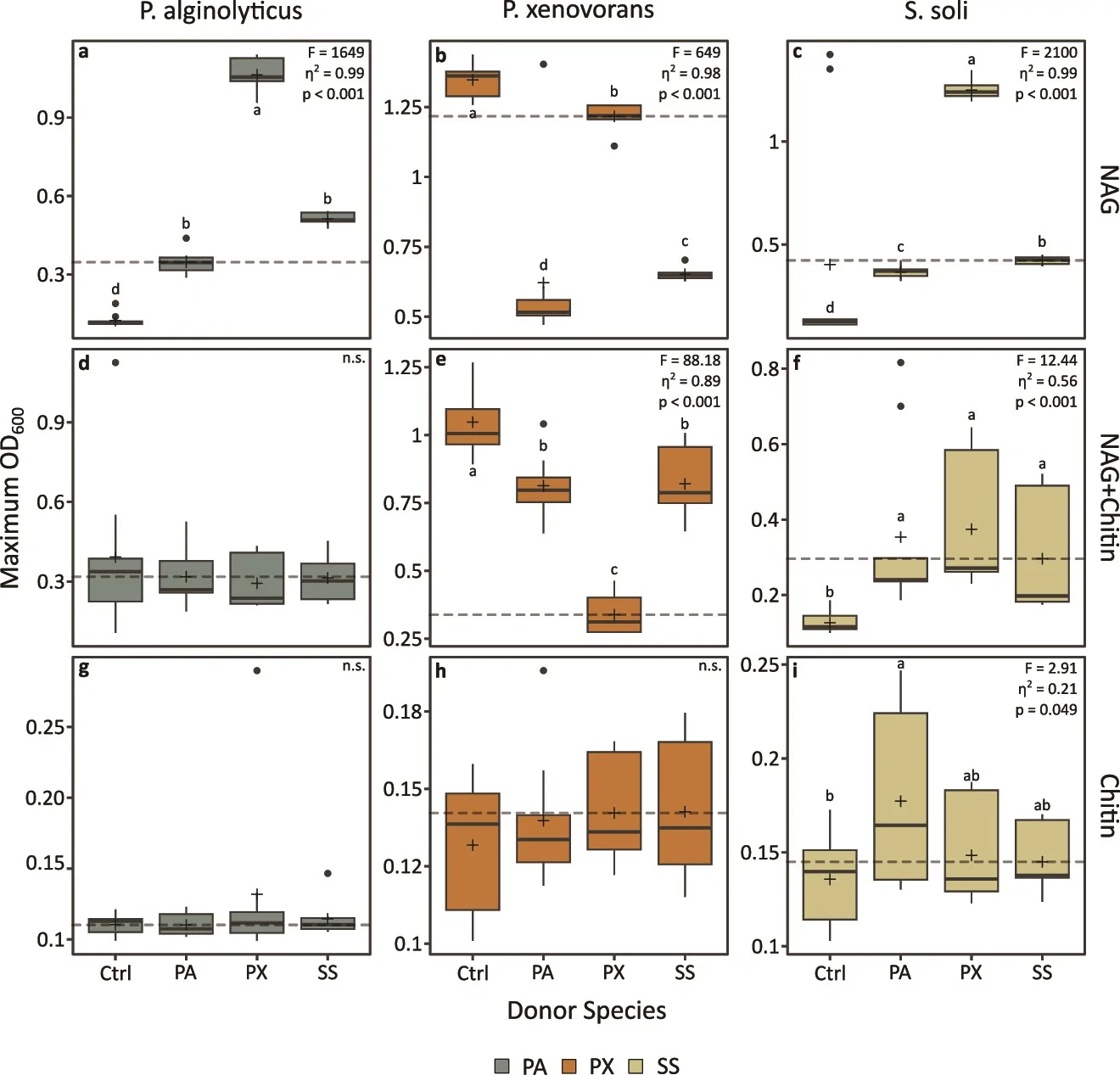
Metabolic interactions. Signals of metabolic interactions were strongest at labile substrate conditions. Boxplots (median + interquartile range) showing the maximally reached optical density at 600 nm (OD_600_) of the three species growing in monoculture (three biological with three technical replicates each) in filtered fresh media, the CFSM of themselves and each other species (“donor species”). Crosses represent the mean. The dashed line indicates the maximally reached OD_600_ of each species growing in its own waste, to serve as a reference for the maximally reached OD_600_ in the other spent media. We interpreted biomass differences between one’s own and another’s spent medium as signals for metabolic interactions, unless biomass was significantly lower in own than in fresh media (i.e. PX in panel e), which hints to substrate depletion in the first round of growth. ANOVA results are indicated on each panel, lowercase letters represent significant differences between different media. Ctrl = control (fresh, previously uninoculated substrate).

NAG supernatants elicited the strongest growth responses of species to the spent media of others (η^2^ = 0.98–0.99), followed by NAG+chitin, while chitin alone showed only weak or non-significant effects. PX’s NAG-waste strongly stimulated PA and SS (Fig. 4a and c), whereas PX itself grew markedly less in their waste (Fig. 4b). In NAG+chitin, PX’s biomass was significantly reduced when growing in its own waste compared to other species’ waste, the fresh medium, and its “own-waste” in the NAG-only treatment. This suggests that PX depleted NAG in the NAG+chitin treatment (containing only half as much NAG as the NAG-only treatment) to growth-limiting levels, while PA or SS did not (comparable to the NAG depletion during the Microresp experiment, S7). This depletion confounded potential metabolic signals, turning the strong negative signal of PA and SS on PX observed in the NAG-only treatment into a seemingly positive signal. The strong positive responses of SS and PA to PX waste weakened in NAG+chitin (Fig. 4d and f) which could also result from nutrient depletion in PX waste. Overall, metabolic interactions were substrate-dependent, with pronounced cross-feeding and inhibition effects emerging primarily in NAG-derived supernatants, while chitin-supported interactions were weak or absent.

### Consumer-resource modeling to resolve interactions

To disentangle basic resource-based effects on respiration of the species combinations, we parametrized a baseline consumer-resource model to the monocultures and compared simulations of duo- and tri-cultures to measured cumulative respiration data. This resulted in a reasonable fit between model prediction and experimental data in the monocultures. However, it could not capture the flattening of the cumulative respiration curves, which might be due to a decrease in biomass after a certain point, or a metabolic change (respiration per unit of biomass) when microbial growth shifts from exponential to stationary phase [47]. As the latter is common in microbial cultures and we do not assume biomass decline right after exponential growth, we attribute curve flattening to a metabolic change at maximum biomass, which the model does not account for. The simulation of PX in NAG+chitin resulted in NAG depletion toward the end of the incubation time, consistent with our measurements (S7). In the model, this resulted in biomass collapse and stagnation of respiration (Fig. 5i), which was less pronounced in the measured data, possibly due to storage compounds or resource recycling. Despite these differences, the model captured the basic growth dynamics of the mono-cultures, so we used it as a tool to predict growth and respiration of co-cultures based on competition for NAG and sharing of decomposition products.

**Figure 5:**
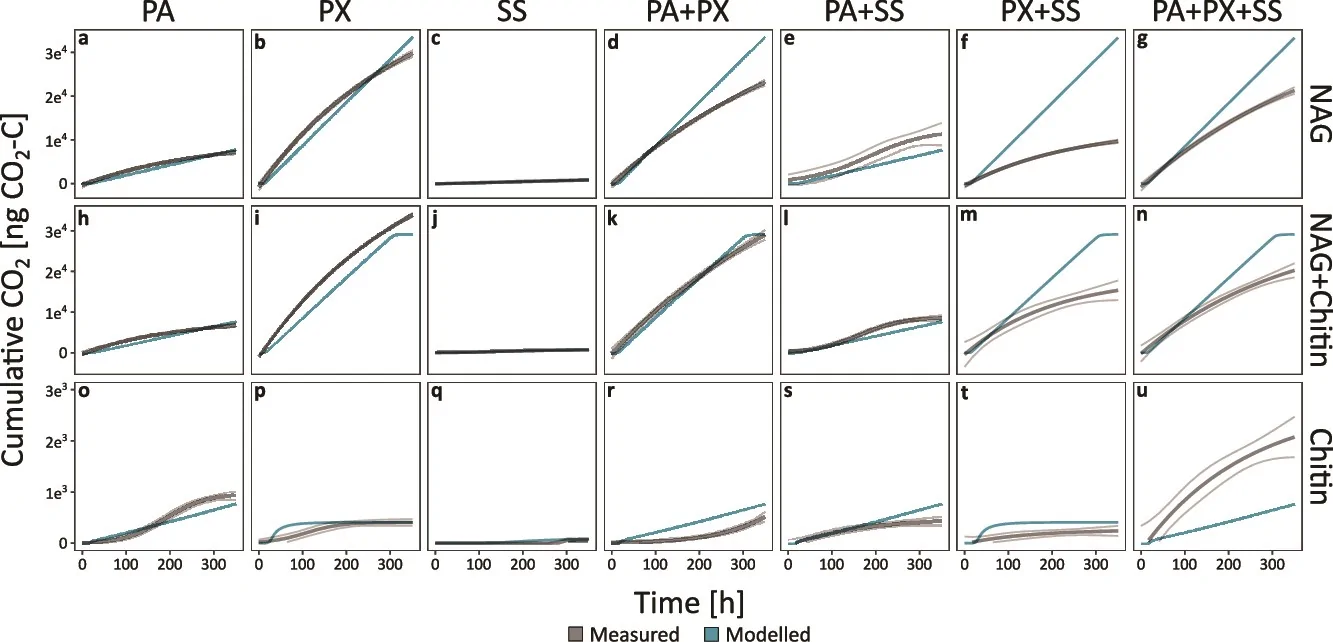
Modeled and measured respiration. Measured cumulative respiration of duo- and tri-cultures differ from model predictions, hinting at interactions beyond those based on primary-resource competition and sharing implemented in the model (S6.2b). Panels show the comparison of measured (best fitting NLS with 95% confidence interval, see Fig. 1) and simulated cumulative respiration over time by a minimal ordinary differential equation-based consumer-resource model, parameterized to the mono-cultures. A schematic of the model, the differential equations, parameters and initial conditions for the different scenarios are provided in S6.

While simulations fit some co-cultures well, they also showed discrepancies, indicating the presence of additional interactions not included in the model. For example, it predicts rapid extinction of PA when in combination with PX in NAG (S6.7), which is not observed in the experiment, where we found increased abundance of PA compared to its mono-culture (Fig. 2a and b). As a result, the model overestimates respiration in this duo-culture (Fig. 5d), reflecting those of the PX mono-culture. In the model, PA only survives in PA + PX duo-cultures in chitin. In this scenario, respiration is overestimated compared to the experiment (Fig. 5r), mirroring the PA mono-culture. Another discrepancy is observed for the PX + SS combination (Fig. 5f, m, and  t), where although SS is not present in the final culture, it appears to exert a negative effect on PX in the experiment, which is not showing up in the model.

In the tri-culture, the model overestimates respiration under NAG and NAG+chitin, again predicting strong competitive effects of PX on the other species. However, in the chitin treatment it vastly underestimates trio respiration. Here, the experimental data suggests facilitative effects if all three species are present, which go beyond the respiration of each of the monocultures and cannot be explained by simple resource-based interactions in the trio-simulation of the model.

### Summary of derived interactions

To quantify the effect of substrate complexity on the nature of interactions, we summarized pairwise interactions between species across substrate treatments based on differences in respiration and final biomass between mono- and duo-cultures and potential metabolic interactions (Fig. 6a) according to rules outlined in the supplement (S8.8) and Fig. 6 caption. Note that we refrained from assessing metabolic interactions in the NAG+chitin treatment as we cannot differentiate them from substrate depletion effects. The observed interactions in NAG and NAG+chitin were similar (and in both cases we see 83.3% of total realized interactions, Fig. 6b) but were very different for chitin (66.7%). Final interaction types combined both directions: positive–positive or neutral–positive were classed as positive; negative–negative or mixed (exploitative) as negative [8]. Expressing the number of interactions of each type in relative to the total number of found interactions according to our ruleset, positive interactions increased with increasing substrate complexity (from 11.1% in NAG to 16.7% in NAG+chitin to 71.4% in chitin) while negative interactions declined (Fig. 6b). Note that the exact values depend on interaction definition according to our ruleset and are therefore only meaningfully compared across substrates within this study.

**Figure 6:**
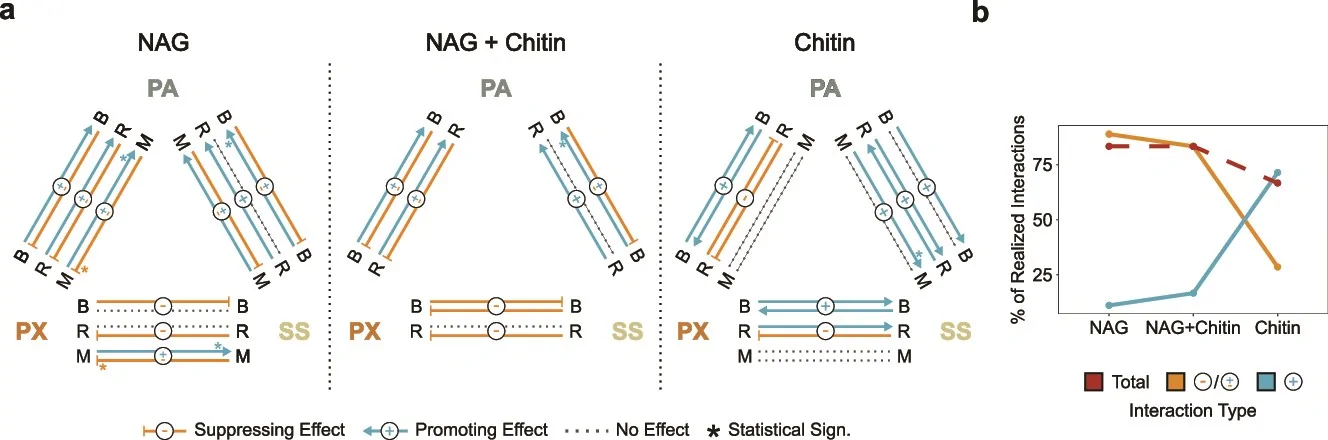
Summary of interactions. The proportion of positive interactions determined from a defined set of rules increased with substrate complexity. Effects of one species on another were derived for the duo-cultures for biomass (B—for SS: endpoint detectability; for PA and PX: more than 10% abundance difference compared to mono-culture, higher = positive, lower = negative effect), cumulative respiration (R—maximum values compared to mono-culture if species was detectable in the duo at the end, higher = positive, lower = negative effect), and maximum growth on metabolites (M—maximum growth compared to its own waste, higher = positive, lower = negative effect) for all three substrate treatments (a). Criteria for effect assignments are described in more detail in the supplement (S8.8). Note that for NAG+chitin, metabolic interactions were not assessed as we could not differentiate them from the effect of previous substrate consumption in the CFSM experiment here (see Fig. 4). An overall positive (+) interaction was assigned if both effects were positive or one was positive, one neutral. Conversely, a negative (−) interaction was assigned in the opposite case (both or one effects negative). A mixed interaction (±) was assigned when one effect was positive and the other negative. For each substrate, the interaction types were expressed in % of the total number of interactions with an effect (b). In addition, the percentage of these measured interactions in relation to the realized interactions is shown (“Total”). Note that this proportion depends on the applied ruleset and are meant as a quantification of the observed trend, not absolute values transferable to other studies.

## Discussion

Our results suggest that this simple community of three chitin-degrading bacterial species exhibits context-dependent interaction patterns that shift with substrate complexity. Based on previous research on social dynamics in degrader communities, we expected to find more positive interactions in the chitin-environment and more negative interactions in NAG. Decreasing the proportion of NAG indeed had a positive effect on the endpoint abundance of less competitive species and decreased the proportion of observable negative interactions according to our ruleset (S8.8). We show that, while negative interactions still dominated, this can already be visible when half of the available carbon is in complex form, suggesting its relevance for real ecosystems where such conditions are common. Putting all three species together increased total biomass, potentially creating a new niche through cross-feeding and thereby enabling more efficient resource utilization. On chitin, tri-culture respiration was significantly higher compared to duo-cultures, suggesting facilitative effects emerging from the presence of all three species, which were unexpected based on duo-culture interactions, and could also not be predicted by the baseline consumer-resource model with simple primary resource-driven interactions.

Based on competition for initially present and enzymatically produced NAG, the model generally predicts competitive exclusion. This often leads to respiration curves closely resembling those of the dominant species, which overestimates the measured values, particularly when species co-exist in the experiment. In contrast, simulations vastly underestimate respiration in the tri-culture on chitin, failing to capture key synergistic interactions. Note that the model assumes that respiration per unit of biomass is constant. Respiration can however also be affected by metabolic or growth-strategy adaptations, changing the amount of respired carbon per unit of biomass [47–50]. Additional data (e.g. time-resolved biomass or transcriptome measurements) would be required to distinguish whether changes in respiration arise from shifts in community composition or from physiological responses. However, we argue that both constitute interactions in their own right, when brought on by the presence of another species, which the model fails to capture. Although it would in principle be possible to extend the model to include additional mechanisms, doing so without additional data could risk overfitting. Future work in this direction would be required to incorporate more elaborate resource-based mechanisms like dynamic metabolism, cross-feeding, necromass recycling, or oxidative enzymes.

We can map functional roles onto the three species based on their observed differences in growth, respiration, and substrate use: SS showed little activity on the original substrates but surprisingly thrived on metabolites produced by others, suggesting a scavenging role. Still, it failed to establish itself in combinations when NAG and with that metabolites were present, implying some sort of competitive suppression of SS when in direct cell–cell contact with the others. PX was the strongest NAG competitor, exhibiting the highest abundance and respiration in NAG treatments but no chitinase activity, suggesting an exploiter role. Its minor respiration on chitin is attributable to residual NAG [13] (S6.3). PA acted as a degrader, producing chitinases, utilizing both substrates and exhibiting the highest respiration of the three on chitin. The model parametrization supports this functional role assignment, suggesting that PX is a copiotroph, thriving in environments with high NAG concentrations, while the uptake system of PA is more specialized to low-NAG conditions, providing a competitive advantage there consistent with its degrader role (S6.6).

Pairwise interactions between species changed from labile to complex substrate, with the proportion of positive interactions increasing, confirming previously observed patterns [17, 18, 20, 21]. SS got outcompeted in all NAG-containing treatments when combined with PA or PX. However, its initial presence still affected the other species: it increased respiration and final biomass of PA and decreased respiration and final biomass of PX compared to their respective mono-cultures. Both effects were not captured by the model, indicating species-specific interactions beyond basic resource competition. The only scenario where SS was still detectable was in the chitin-only treatment, potentially indicating a shift from a mostly exploitative (with PA) or negative (with PX) relationship to one also benefitting SS. While total respiration in the PA + SS duo-culture was reduced compared to the PA mono-culture in chitin, earlier onset suggests initial positive effects. Although PX always outcompeted SS when NAG was present, it had a positive effect on PA, likely by PA cross-feeding on PX’s metabolites in NAG.

Substrate use shifted in the face of competitive pressure, potentially explaining co-occurrence of competing species [51, 52]. In NAG+chitin, assessed interactions were similar to NAG, with a slightly increased proportion of positive interactions given our ruleset. Total respiration and PA’s final relative abundance were increased compared to NAG, the latter also visible in the tri-culture. Additionally, predicted respiration better matched measured respiration compared to the overestimation in NAG, suggesting alleviation of suppressing interactions when chitin was added. Possibly, the presence of chitin as another alternative substrate expanded PA’s resource-use niche [17, 21, 53], from which PX could have also profited. Removing NAG entirely further increased PA’s relative abundance but delayed respiration, potentially because competition for limited amounts of readily available substrate in the chitin-only treatment suppressed overall activity.

The initial presence of all three species increased total final biomass across treatments, even in NAG. Although SS again was not detectable, biomass of both PA and PX were increased compared to their duo-cultures in the NAG-containing treatments (and higher than their mono-cultures in NAG-only), while respiration was similar. This could be explained by metabolic cross-feeding, which can increase overall substrate-use efficiency [54] by more complete carbon utilization [3, 21], reducing respiration compared to biomass [55]. Additionally, necromass recycling could have similar effects [56]. As shown in our spent-media experiment, PA and SS benefit from PX metabolites when grown in NAG. This did not translate to increased fitness for SS in the main experiment, though it likely did for PA. For NAG+chitin, cross-feeding signals were weaker and confounded by resource depletion in the spent-media assay, but we still observed a similar respiration and biomass pattern. Beyond cross-feeding, the transient presence of SS measurably altered PA and PX dynamics in the tri-culture, an effect not predictable from duo-culture results alone [23, 24]. Note that we only measured a final snapshot of the cell abundances, and differences between treatments could also reflect different temporal growth patterns of species in different conditions. However, as peak activity, likely marking the transition from exponential to stationary growth, occurred at similar times (S4), we believe the community compositions we detected at the endpoint are comparable. As mentioned before, our lack of time-resolved biomass data prevents us from observing transient dynamics directly and the detectability with our approach may also reflect the presence of residual DNA instead of viable cells. Future investigations should therefore be complemented with viability assays to give a better idea of stable co-existence in the final communities. Generally, however, our findings show that increasing the species number to three had a positive effect on the system, in line with ecological theory on diversity-ecosystem functioning [57, 58].

The interplay of substrate complexity and the number of species cumulated in the tri-culture activity on chitin, where we observed the most even final community and the highest respiration and final biomass of this substrate. Interestingly, classical division of labor in enzyme production was likely absent in our system as PA was the sole chitin degrader. Oxidative enzymes produced by PX (S3) could in theory increase decomposition efficiency [59], however we did not obtain direct evidence for this. SS was not entirely outcompeted but still detectable at the end. This possibly increased the fitness of PA, increasing community-wide carbon supply and resulting in the earlier onset and higher respiration compared to the mono- or duo-cultures and model prediction. The presence of all three species alleviated the constraints observed in duo-culture, especially between PX and PA, where productivity on chitin was delayed by high competitive pressure and low labile substrate availability. Synergistic interactions and cascading effects have been shown to be an integral part of more diverse complex substrate-degrading communities [3, 13, 15, 16, 55]. Our study underlines that this can already be seen with the low species richness of three. It is of interest that, in contrast to most previous studies, this consortium did not assemble in a natural context and thus shares no co-evolutionary history that could explain observed synergies.

While overall interaction directions defined by our ruleset were similar between NAG and NAG+chitin, suggesting that the effect of NAG on interactions is stronger than the effect of chitin, we observed that alleviation of competition can already emerge when half of the available carbon is in complex form (see the higher relative abundance of PA in PA + PX NAG+chitin), and that the proportion relative to the total assessed interactions according to our ruleset was slightly increased. It is important to note here that the absolute proportions of interactions depend on these rules, however we assume the overall qualitative pattern would remain consistent across other definitions. The presence of complex substrates could buffer effects of negative interactions induced by labile substrate addition in diverse communities, preserving overall functioning. To test this more directly, NAG could be added to the tri-culture previously established on chitin. It would be interesting to see whether this would lead to the extinction of SS, possibly with long-term consequences for community functioning once all NAG is depleted again, or whether previously established positive interactions could prevent this.

Taken together, our findings show that substrate complexity promotes facilitative over competitive interactions, particularly as species number increases, with consequences for the functioning of degrader communities. The respiration curves predicted by our parsimonious baseline model also suggest that the implemented competition for and sharing of basic resources (NAG and enzymes) alone cannot fully account for the observed dynamics, which might underpin microbe-driven ecosystem processes in environments where diverse communities decompose complex substrates under fluctuating substrate conditions. We note that our experiments (and most others investigating similar questions [17–19, 21]) were conducted in a well-mixed system, whereas natural soils or marine chitin particles are spatially structured. This fundamentally affects resource availability as public goods are not evenly spread but only subject to diffusion, affecting interactions based on the spatial position of cells. Similarly, labile substrate inputs, for example at root tips, are also spatially constrained. The extent to which our findings translate to spatially structured environments therefore remains to be tested. All in all however, our results support the notion that diverse, but carbon limited environments might naturally foster positive interactions, illustrating that the prevalence of positive microbial interactions may be higher than previously appreciated. As noted before [19], this study however also underscores that the community response to labile substrate addition depends on the diversity and properties of the involved species, and shows that decomposition outcomes inferred from single-substrate or pairwise assays may not extrapolate to complex-substrate degrading communities, where both substrate availability and the number of co-occurring species shape the prevailing interactions. Recognizing these context-dependent interactions will be crucial for predicting how microbial communities regulate carbon turnover and ecosystem stability.

## Supplementary Material

Supplement_ISME_comm_ycag272

## Data Availability

The datasets generated during and/or analysed during the current study are available at Zenodo: https://doi.org/10.5281/zenodo.18483933.

## References

[1] Kehe J, Ortiz A, Kulesa A et al. Positive interactions are common among culturable bacteria. *Sci Adv* 2021;7:eabi7159. 10.1126/sciadv.abi7159 PMC8570599 34739314

[2] Giri S, Waschina S, Kaleta C et al. Defining division of labor in microbial communities. *J Mol Biol* 2019;431:4712–31. 10.1016/j.jmb.2019.06.023 31260694

[3] McClure R, Farris Y, Danczak R et al. Interaction networks are driven by community-responsive phenotypes in a chitin-degrading consortium of soil microbes. *mSystems* 2022;7:e00372–22. 10.1128/msystems.00372-22 PMC9599572 36154140

[4] Oliveira NM, Niehus R, Foster KR. Evolutionary limits to cooperation in microbial communities. *Proc Natl Acad Sci* 2014;111:17941–6. 10.1073/pnas.1412673111 PMC4273359 25453102

[5] Thommes M, Wang T, Zhao Q et al. Designing metabolic division of labor in microbial communities. *mSystems* 2019;4:e00263–18. 10.1128/mSystems.00263-18 PMC6456671 30984871

[6] Ratzke C, Barrere J, Gore J. Strength of species interactions determines biodiversity and stability in microbial communities. *Nat Ecol Evol* 2020;4:376–83. 10.1038/s41559-020-1099-4 32042124

[7] Coyte KZ, Schluter J, Foster KR. The ecology of the microbiome: networks, competition, and stability. *Science* 2015;350:663–6. 10.1126/science.aad2602 26542567

[8] Palmer JD, Foster KR. Bacterial species rarely work together. *Science* 2022;376:581–2. 10.1126/science.abn5093 35511986

[9] Pazos F, Valencia A, De Lorenzo V. The organization of the microbial biodegradation network from a systems-biology perspective. *EMBO Rep* 2003;4:994–9. 10.1038/sj.embor.embor933 PMC1326392 12973298

[10] Pollak S, Gralka M, Sato Y et al. Public good exploitation in natural bacterioplankton communities. *Sci Adv* 2021;7:eabi4717. 10.1126/sciadv.abi4717 PMC8318375 34321201

[11] Beier S, Bertilsson S. Bacterial chitin degradation-mechanisms and ecophysiological strategies. *Front Microbiol* 2013;4:149. 10.3389/fmicb.2013.00149 PMC3682446 23785358

[12] Pelz O, Tesar M, Wittich RM et al. Towards elucidation of microbial community metabolic pathways: unravelling the network of carbon sharing in a pollutant-degrading bacterial consortium by immunocapture and isotopic ratio mass spectrometry. *Environ Microbiol* 1999;1:167–74. 10.1046/j.1462-2920.1999.00023.x 11207732

[13] Pontrelli S, Szabo R, Pollak S et al. Metabolic crossfeeding structures the assembly of polysaccharide degrading communities. *Sci Adv* 2022;8:eabk3076. 10.1126/sciadv.abk3076 PMC8865766 35196097

[14] Kaiser C, Franklin O, Richter A et al. Social dynamics within decomposer communities lead to nitrogen retention and organic matter build-up in soils. *Nat Commun* 2015;6:8960. 10.1038/ncomms9960 PMC4697322 26621582

[15] Datta MS, Sliwerska E, Gore J et al. Microbial interactions lead to rapid micro-scale successions on model marine particles. *Nat Commun* 2016;7:11965. 10.1038/ncomms11965 PMC4915023 27311813

[16] Enke TN, Datta MS, Schwartzman J et al. Modular assembly of polysaccharide-degrading marine microbial communities. *Curr Biol* 2019;29:1528–1535.e6. 10.1016/j.cub.2019.03.047 31031118

[17] Abdoli P, Vulin C, Lepiz M et al. Substrate complexity buffers negative interactions in a synthetic community of leaf litter degraders. *FEMS Microbiol Ecol* 2024;100:fiae102. 10.1093/femsec/fiae102 PMC11289631 39020097

[18] Cortes-Tolalpa L, Salles JF, Van Elsas JD. Bacterial synergism in lignocellulose biomass degradation—complementary roles of degraders as influenced by complexity of the carbon source. *Front Microbiol* 2017;8:1628. 10.3389/fmicb.2017.01628 PMC5641323 29067002

[19] Deng YJ, Wang SY. Synergistic growth in bacteria depends on substrate complexity. *J Microbiol* 2016;54:23–30. 10.1007/s12275-016-5461-9 PMC4822414 26727898

[20] Deng YJ, Wang SY. Complex carbohydrates reduce the frequency of antagonistic interactions among bacteria degrading cellulose and xylan. *FEMS Microbiol Lett* 2017;364:fnx019. 10.1093/femsle/fnx019 PMC5974935 28130369

[21] McNichol SM, Sanchez-Quete F, Loeb SK et al. Dynamics of carbon substrate competition among heterotrophic microorganisms. *ISME J* 2024;18:wrae018. 10.1093/ismejo/wrae018 PMC10942773 38366177

[22] Friedman J, Higgins LM, Gore J. Community structure follows simple assembly rules in microbial microcosms. *Nat Ecol Evol* 2017;1:0109. 10.1038/s41559-017-0109 28812687

[23] Levine JM, Bascompte J, Adler PB et al. Beyond pairwise mechanisms of species coexistence in complex communities. *Nature* 2017;546:56–64. 10.1038/nature22898 28569813

[24] Ishizawa H, Tashiro Y, Inoue D et al. Learning beyond-pairwise interactions enables the bottom–up prediction of microbial community structure. *Proc Natl Acad Sci* 2024;121:e2312396121. 10.1073/pnas.2312396121 PMC10873592 38315845

[25] Garcia Arredondo M, Kew W, Chu R et al. Differential exudation creates biogeochemically distinct microenvironments during rhizosphere evolution. *Environ Sci Technol* 2024;58:18713–22. 10.1021/acs.est.4c04108 39390789

[26] Wiesenbauer J, König A, Gorka S et al. A pulse of simulated root exudation alters the composition and temporal dynamics of microbial metabolites in its immediate vicinity. *Soil Biol Biochem* 2024;189:109259. 10.1016/j.soilbio.2023.109259

[27] Hu Y, Wang Z, Wang Q et al. Climate change affects soil labile organic carbon fractions in a Tibetan alpine meadow. *J Soils Sediments* 2017;17:326–39. 10.1007/s11368-016-1565-4

[28] Phillips RP, Finzi AC, Bernhardt ES. Enhanced root exudation induces microbial feedbacks to N cycling in a pine forest under long-term CO_2_ fumigation. *Ecol Lett* 2011;14:187–94. 10.1111/j.1461-0248.2010.01570.x 21176050

[29] Richardson CM, Peucker-Ehrenbrink B, Wyatt S et al. Effects of climate change on river and groundwater nutrient inputs to the coastal ocean. *Commun Earth Environ* 2025;6:761. 10.1038/s43247-025-02594-6

[30] Zhang H, Lauerwald R, Ciais P et al. Global changes alter the amount and composition of land carbon deliveries to European rivers and seas. *Commun Earth Environ* 2022;3:245. 10.1038/s43247-022-00575-7

[31] Patowary R, Deka H. Chapter 17—Paenibacillus. In: Amaresan N., Senthil Kumar M., Annapurna K. et al. (eds.), Beneficial Microbes in Agro-Ecology. London: Academic Press, 2020, 339–61. 10.1016/B978-0-12-823414-3.00017-4

[32] Bako CM, Mattes TE, Marek RF et al. Biodegradation of PCB congeners by Paraburkholderia xenovorans LB400 in presence and absence of sediment during lab bioreactor experiments. *Environ Pollut* 2021;271:116364. 10.1016/j.envpol.2020.116364 PMC8183161 33412450

[33] Kim MK, Na JR, Lee TH et al. Solirubrobacter soli sp. nov., isolated from soil of a ginseng field. *Int J Syst Evol Microbiol* 2007;57:1453–5. 10.1099/ijs.0.64715-0 17625174

[34] Caspi R, Billington R, Keseler IM et al. The MetaCyc database of metabolic pathways and enzymes—a 2019 update. *Nucleic Acids Res* 2020;48:D445–53. 10.1093/nar/gkz862 PMC6943030 31586394

[35] Karp PD, Midford PE, Billington R et al. Pathway tools version 23.0 update: software for pathway/genome informatics and systems biology. *Brief Bioinform* 2021;22:109–26. 10.1093/bib/bbz104 PMC8453236 31813964

[36] Souza CP, Burbano-Rosero EM, Almeida BC et al. Culture medium for isolating chitinolytic bacteria from seawater and plankton. *World J Microbiol Biotechnol* 2009;25:2079–82. 10.1007/s11274-009-0098-z

[37] Dashti AA, Jadaon MM, Abdulsamad AM et al. Heat treatment of bacteria: a simple method of DNA extraction for molecular techniques. *Kuwait Med J* 2009;41:117–22.

[38] Pjevac P, Hausmann B, Schwarz J et al. An economical and flexible dual barcoding, two-step PCR approach for highly multiplexed amplicon sequencing. *Front Microbiol* 2021;12:669776. 10.3389/fmicb.2021.669776 PMC8173057 34093488

[39] McMurdie PJ, Holmes S. Phyloseq: an R package for reproducible interactive analysis and graphics of microbiome census data. *PLoS One* 2013;8:e61217. 10.1371/journal.pone.0061217 PMC3632530 23630581

[40] Martin V, Schmidt H, Canarini A et al. Soil cover shapes organic matter pools and microbial communities in soils of maritime Antarctica. *Geoderma* 2024;446:116894. 10.1016/j.geoderma.2024.116894

[41] Alteio LV, Séneca J, Canarini A et al. A critical perspective on interpreting amplicon sequencing data in soil ecological research. *Soil Biol Biochem* 2021;160:108357. 10.1016/j.soilbio.2021.108357

[42] Choi Y, Jeong J, Kim M et al. Backtracking identification techniques for predicting unclear bacterial taxonomy at species level: molecular diagnosis-based bacterial classification. *Genes Genomics* 2025;47:503–8. 10.1007/s13258-025-01634-x 40111647

[43] Salas E, Gorfer M, Bandian D et al. A rapid and sensitive assay to quantify amino sugars, neutral sugars and uronic acid necromass biomarkers using pre-column derivatization, ultra-high-performance liquid chromatography and high-resolution mass spectrometry. *Soil Biol Biochem* 2023;177:108927. 10.1016/j.soilbio.2022.108927

[44] Georgiou K, Abramoff RZ, Harte J et al. Microbial community-level regulation explains soil carbon responses to long-term litter manipulations. *Nat Commun* 2017;8:1223. 10.1038/s41467-017-01116-z PMC5663850 29089496

[45] Hilau S, Katz S, Wasserman T et al. Density-dependent effects are the main determinants of variation in growth dynamics between closely related bacterial strains. *PLoS Comput Biol* 2022;18:e1010565. 10.1371/journal.pcbi.1010565 PMC9578580 36191042

[46] Ter Braak CJF, Vrugt JA. Differential evolution Markov chain with snooker updater and fewer chains. *Stat Comput* 2008;18:435–46. 10.1007/s11222-008-9104-9

[47] Riedel TE, Berelson WM, Nealson KH et al. Oxygen consumption rates of bacteria under nutrient-limited conditions. *Appl Environ Microbiol* 2013;79:4921–31. 10.1128/AEM.00756-13 PMC3754705 23770901

[48] Cakin I, Millington R, Pawar S et al. A novel method to simultaneously estimate bacterial respiration and growth from oxygen dynamics. *ISME Commun* 2026;6:ycag024. 10.1093/ismeco/ycag024 PMC12963047 41798180

[49] Hagerty SB, Allison SD, Schimel JP. Evaluating soil microbial carbon use efficiency explicitly as a function of cellular processes: implications for measurements and models. *Biogeochemistry* 2018;140:269–83. 10.1007/s10533-018-0489-z

[50] Lipson DA . The complex relationship between microbial growth rate and yield and its implications for ecosystem processes. *Front Microbiol* 2015;6:615. 10.3389/fmicb.2015.00615 PMC4468913 26136742

[51] Bauer MA, Kainz K, Carmona-Gutierrez D et al. Microbial wars: competition in ecological niches and within the microbiome. *Microb Cell* 2018;5:215–9. 10.15698/mic2018.05.628 PMC5961915 29796386

[52] Ghoul M, Mitri S. The ecology and evolution of microbial competition. *Trends Microbiol* 2016;24:833–45. 10.1016/j.tim.2016.06.011 27546832

[53] Lindemann SR . A piece of the pie: engineering microbiomes by exploiting division of labor in complex polysaccharide consumption. *Curr Opin Chem Eng* 2020;30:96–102. 10.1016/j.coche.2020.08.004 PMC7505235 32968619

[54] Douglas AE . The microbial exometabolome: ecological resource and architect of microbial communities. *Philos Trans R Soc B Biol Sci* 2020;375:20190250. 10.1098/rstb.2019.0250 PMC7133521 32200747

[55] McClure R, Garcia M, Couvillion S et al. Removal of primary nutrient degraders reduces growth of soil microbial communities with genomic redundancy. *Front Microbiol* 2023;13:1046661. 10.3389/fmicb.2022.1046661 PMC9902710 36762098

[56] Hao YQ, Li BH, Chen JY et al. Bacterial necromass recycling promotes diversity maintenance in bacterial communities via resource partitioning. *Nat Ecol Evol* 2026;10:580–93. 10.1038/s41559-025-02967-2 41606115

[57] Becker J, Eisenhauer N, Scheu S et al. Increasing antagonistic interactions cause bacterial communities to collapse at high diversity. *Ecol Lett* 2012;15:468–74. 10.1111/j.1461-0248.2012.01759.x 22394557

[58] Jousset A, Schmid B, Scheu S et al. Genotypic richness and dissimilarity opposingly affect ecosystem functioning. *Ecol Lett* 2011;14:537–45. 10.1111/j.1461-0248.2011.01613.x 21435139

[59] Vaaje-Kolstad G, Westereng B, Horn SJ et al. An oxidative enzyme boosting the enzymatic conversion of recalcitrant polysaccharides. *Science* 2010;330:219–22. 10.1126/science.1192231 20929773

