# Supplementary material for: Substrate complexity and species number jointly shift the balance between competition and facilitation in a chitin-degrading bacterial consortium
Source: ISME Commun. 2026 Sep 25;6(1):ycag272. doi: 10.1093/ismeco/ycag272 (PMC13662801; doi:10.1093/ismeco/ycag272)
Supplement: Supplementary file 1 [file supplement_isme_comm_ycag272.docx]

Substrate complexity and species number jointly shift the balance between competition and facilitation in a chitin-degrading bacterial consortium

Supplementary Material

Moritz Mohrlok^1,2^, Lauren Alteio^3^, Julia Berger^1^, Ksenia Guseva^1^, Erika Salas^1^, Monika Schmoll^1^, Dave Sirbu^1^ and Christina Kaiser^1^

^1^University of Vienna, Centre for Microbiology and Environmental Systems Science, Department for Microbiology and Ecosystem Science, Division of Terrestrial Ecosystem Research, Vienna, Austria.

^2^University of Vienna, Doctoral School in Microbiology and Environmental Science, Vienna, Austria

^3^Austrian Competence Centre for Feed and Food Quality, Safety and innovation, FFoQSI GmbH, Tulln, Austria.

S1: Statistical Results

S1.1: Two-way ANOVA total respiration

| Homogeneity of variance | | | ANOVA | | | | Normality of residuals | | Effect size |
| --- | --- | --- | --- | --- | --- | --- | --- | --- | --- |
| df | F | p | Factor | df | F | p | W | p | η2 |
| 2 | 0.096 | 0.909 | Species | 6 | 22.618 | < 0.001 | 0.96 | 0.112 | 0.15 |
|  |  |  | Substrate | 2 | 337.526 | < 0.001 |  |  | 0.75 |
|  |  |  | Interaction | 11 | 5.718 | < 0.001 |  |  | 0.07 |

Full results of a two-way ANOVA testing the effect of species and substrate on the total amount of respired C in each treatment, corresponding to Figure 1. Data was log-transformed and the minimal respiration of *S. soli* growing on chitin had to be removed to meet ANOVA assumptions. Homogeneity of variance was tested with Levene’s test and normality of residuals was checked with Shapiro-Wilk’s test. Df = degrees of freedom, F = F-value, p = p-value (threshold α = 0.05), W = Shapiro-Wilk test statistic.

S1.2: Null model comparison nonlinear regressions fit to respiration data

|  | NAG | | | |  | NAG+Chitin | | | |  | Chitin | | | |  |
| --- | --- | --- | --- | --- | --- | --- | --- | --- | --- | --- | --- | --- | --- | --- | --- |
|  | RSE | df | F | p | acf | RSE | df | F | p | acf | RSE | df | F | p | acf |
| PA | 586.7 | 45 | 332.28 | <0.001 | 0.39 | 734.9 | 29 | 433.93 | <0.001 | 0.22 | 1043 | 45 | 7.83 | <0.001 | 0.35 |
| PX | 1033 | 45 | 106.74 | <0.001 | 0.28 | 2704 | 29 | 531.07 | <0.001 | 0.34 | 612.5 | 45 | 5.38 | <0.001 | 0.23 |
| SS | 106.1 | 29 | 8334.5 | <0.001 | 0.19 | 293.7 | 13 | 39711 | <0.001 | 0.13 | 553.8 | 45 | 125.61 | <0.001 | 0.33 |
| PA+PX | 51.29 | 13 | 58.4 | <0.001 | 0.32 | 2935 | 29 | 31.18 | <0.001 | 0.15 | 129 | 45 | 6.5 | <0.001 | 0.36 |
| PA+SS | 1277 | 29 | 3.69 | <0.001 | 0.33 | 1978 | 29 | 190.2 | <0.001 | 0.12 | 25.48 | 29 | 1.8 | 0.0225 | 0.08 |
| PX+SS | 445.7 | 13 | 269.34 | <0.001 | 0.19 | 108.7 | 45 | 5.35 | <0.001 | 0.15 | 70.41 | 29 | 1.68 | n.s. | 0.08 |
| PA+PX+SS | 130 | 45 | 55.89 | <0.001 | 0.37 | 128.2 | 29 | 12.53 | <0.001 | 0.30 | 582.3 | 45 | 180.7 | <0.001 | 0.28 |

Full F-test results corresponding to Figure 1 of comparing each fitted nonlinear regression to a null model containing all data in a substrate/biodiversity treatment combination (e.g. NAG/duo). For the trio-cultures, this was tested across all three substrate treatments. P-values were corrected for multiple testing (Benjamini-Hochberg). Column “acf” shows the mean autocorrelation value across the entire timeseries for each fit. RSE = Residual standard error, df = degrees of freedom, F = F-value, p = P-value (α = 0.05). PA = *Paenibacillus alginolyticus*, PX = *Paraburkholderia xenovorans*, SS = *Solirubrobacter soli*.

S1.3: Two-Way ANOVA results final community (total and within each species)

| Species | Homogeneity of variance | | | ANOVA | | | | Normality of residuals | | Effect size |
| --- | --- | --- | --- | --- | --- | --- | --- | --- | --- | --- |
|  | df | F | p | Factor | df | F | p | W | p | η2 |
| Total | 20 | 0.740 | 0.750 | Species combination | 6 | 36.96 | < 0.001 | 0.954 | 0.08 | 0.46 |
|  |  |  |  | Substrate | 2 | 57.69 | < 0.001 |  |  | 0.24 |
|  |  |  |  | Interaction | 12 | 10.03 | < 0.001 |  |  | 0.25 |
| PA | 15 | 0.816 | 0.653 | Species combination | 3 | 12.53 | < 0.001 | 0.942 | 0.04 | 0.2 |
|  |  |  |  | Substrate | 3 | 19.75 | < 0.001 |  |  | 0.32 |
|  |  |  |  | Interaction | 9 | 7.11 | < 0.001 |  |  | 0.35 |
| PX | 15 | 0.407 | 0.961 | Species combination | 3 | 7.27 | 0.001 | 0.946 | 0.07 | 0.06 |
|  |  |  |  | Substrate | 3 | 88.79 | < 0.001 |  |  | 0.77 |
|  |  |  |  | Interaction | 9 | 4.11 | 0.003 |  |  | 0.11 |
| SS | 9 | 2.01 | 0.122 | Species combination | 3 | 4.10 | 0.035 | 0.927 | 0.119 | 0.14 |
|  |  |  |  | Substrate | 3 | 21.87 | < 0.001 |  |  | 0.67 |
|  |  |  |  | Interaction | 3 | 7.55 | 0.005 |  |  | 0.13 |

Full results of a two-way ANOVA testing the effect of species combination and substrate on the absolute abundance of each target species, corresponding to each color in Figure 2. Data was log-transformed and replicate A of PA+SS growing on chitin and replicate B PX growing in monoculture in NAG and NAG+chitin was removed to meet ANOVA assumptions. A TukeyHSD post-hoc test was performed, significantly different groups are indicated by lowercase letters in Figure 2a. Homogeneity of variance was tested with Levene’s test and normality of residuals was checked with Shapiro-Wilk’s test. Df = degrees of freedom, F = F-value, p = p-value (threshold α = 0.05), W = Shapiro-Wilk test statistic, PA = *Paenibacillus alginolyticus*, PX = *Paraburkholderia xenovorans*, SS = *Solirubrobacter soli*.

S2: Chitin degradation and potential utilization pathways

S2.1: Description of chitin degradation

Chitin is an insoluble, crystalline polymer of β-1,4-linked *N*-acetyl-D-glucosamine (NAG). Natural chitin occurs in three crystalline forms that differ in the orientation of micro-fibrils [1]: The α-form, with antiparallel chains, is the most abundant and most recalcitrant and is the form found in crustacean shells as used in this work and fungal cell walls; the β-form, with parallel chains, is more readily hydrolysed, while the γ-form combines both arrangements. Because the polymer cannot be assimilated directly, its metabolic use requires extracellular hydrolysis prior to uptake, so degradation proceeds as a multi-step cascade [1]. There are two distinct chitinoclastic routes that lead to chitin breakdown. The chitinolytic route proceeds in three steps: chitinases (predominantly glycoside hydrolase family GH18) with different points of attack (e.g. exoenzymes targeting the end of the chains and endoenzymes targeting the middle [2]) first cleave the polymer into oligomers and then into chitobiose by hydrolysing the (1,4)-β-glycosidic bonds, while β-*N*-acetyl-hexosaminidases (GH20) release NAG, generally in the periplasm or cytoplasm [1]. Individual strains frequently produce several chitinases with complementary endo-/exo- and processive/non-processive modes of action, and non-catalytic chitin-binding proteins can disrupt the crystalline surface to improve substrate accessibility [1]. Chitin can also be deacetylated, producing chitosan, and subsequently deaminated, resulting in cellulose, and different sets of enzymes can target these chitin-derived polymers. The second route is oxidative and is initiated by lytic polysaccharide monooxygenases (LPMOs; CAZy auxiliary-activity families AA10, AA11 and AA15). These enzymes oxidize the C1 carbon at the crystalline surface, generating chain breaks that act synergistically with hydrolytic chitinases to accelerate depolymerization [3,4].

S2.2: Potential chitin utilization pathways

|  | *P. alginolyticus* | *P. xenovorans* | *S. soli* |  | |
| --- | --- | --- | --- | --- | --- |
| **EC-#** | **PA** | **PX** | **SS** | **Activity** | **Target Substrate** |
| 3.2.1.14 | - | x | - | Endo-Chitinase | Chitin |
| 3.2.1.200 | - | x | - | Exo-Chitinase | Chitodextrin |
| 3.2.1.200 | - | x | - | Exo-Chitinase | Chitodextrin |
| 3.2.1.52 | x | x | - | Exo-Chitinase | Chitotriose |
| 3.2.1.52 | x | x | - | Exo-Chitinase | Chitobiose |
| 3.2.1.52 | x | x | x | Muramidase | Peptidoglycan |
| 3.2.1.52 | - | x | - | Muramidase | Peptidoglycan |
| 3.2.1.52 | x | x | - | β-Hexosaminidase | N-acetyl-β-D-hexosaminide |
| 3.2.1.52 | x | x | - | β-Hexosaminidase | N-acetyl-β-D-hexosaminide |
| 3.2.1.86 | x | - | x | Chitobiose Metabolism | N-monoacetylchitobiose- 6-phosphate |
| 3.5.99.6 | x | x | x | NAG Metabolism | α-D-glucosamine-6-phosphate |

Results of a MetaCyc (metacyc.org [5]) comparative analysis search for known pathways involved in chitin degradation using the integrated software Pathway Tools [6]), chosen pathway class: “Degradation/utilization/assimilation - carbohydrate degradation”) in the genomes of the three species *P. alginolyticus, P. xenovorans* and *S. soli.* Activity and target substrate information were obtained from the corresponding Pathway Tools reaction (“x” = presence of the pathway, “-“ = absence of the pathway). The results show that each species has the genomic potential to utilize N-acetylglucosamine (NAG) and PA and PX encode for known chitin-active enzymes. *S. soli* has the potential for a muramidase, which can non-specifically degrade chitosan [7], a de-acetylated form of chitin.

S3: Genome screening for carbohydrate active enzymes

|  | *P. alginolyticus* | *P. xenovorans* | *S. soli* |  |  |
| --- | --- | --- | --- | --- | --- |
| **CAZy class** | **PA** | **PX** | **SS** | **Activity** | **Target Substrate** |
| GH18 | 7/3* | 1 | 1* (+2) | Chitinase | Chitin |
| GH19 | 0 | 1 | 1 | Chitinase | Chitin |
| GH3 | 3 | 1 | 1* | NAGase, NAG-phosphorylase | Chitin |
| GH94 | 0 | 2 | 0 | Diacetyl chiotobiose phosphorylases | Chitin |
| CE4 | 0 | 1 | 1* | Chitin deacetylases |  |
| GH46 | 0 | 0 | 0 (1) | Chitosanases |  |
| GH75 | 0 | 0 | 0 (1) | Chitosanases |  |
| CBM50 | 3 (+4) | 6 | 2 | Chitin binding modules | Chitin |
| AA10 | 0 | 1* | 0 | LPMOS | Chitin |
| AA15 | 0 | 0 | 0 (1) | LPMOs |  |
| AA7 | 0 (1) | 1 | 11 | Chitooligosaccharide oxidases | Chitooligosaccharides |

Genomes (obtained from the National Center for Biotechnology Information, NCBI) of the three strains were additionally screened for carbohydrate active enzyme (CAZyme) encoding genes with the tool dbCAN2 [8] in combination with the CUPP (conserved unique peptide patterns) algorithm [9]. This allowed us to find possible unknown enzymes involved in chitin degradation based on their predicted structure and function. Numbers in brackets represent enzymes that are predicted by the CUPP algorithm. Stars indicate enzymes with a secretion signal peptide. NAG = N-acetylglucosamine, LPMO = lytic polysaccharide monooxygenase. The results show that *P. alginolyticus* has the highest potential to produce and secrete conventional chitinases, in accordance with our chitin degradation potential measurements (Fig. 3). *P. xenovorans* is able to produce and secrete an LPMO. It is however unlikely that these enzymes cause the measured respiration of the species on chitin, as these enzymes are generally thought to be auxiliary enzymes synergizing with hydrolytic chitinases [3] that are not able to fully depolymerize the molecule on their own. *S. soli* also encodes for some chitin-active enzymes, but we observed no respiration of the species on chitin nor meaningful extracellular chitinase activity, so our experimental conditions likely did not induce expression of these enzymes.

S4: Respiration Slope

**Figure S4:** Time-derivative (slope) of the cumulative respiration curves (see Figure 1) over time. This was calculated as the change in cumulative respiration at each timepoint compared to its previous one, using the predicted data of the nonlinear least squares regressions fit to the cumulative respiration data as a basis. The data shows that, in most cases, respiration rates peaked around 100-200 hours, likely corresponding to the point with the highest biomass. Most communities were in stationary phase at the end of the experiment.

S5: Starting Community

**Figure S5:** Initial community structure for all species and substrate combinations. The scale is directly comparable to Figure 2. Cell numbers were measured for the monocultures in the same way as for the community data provided in Figure 2 and then estimated for the combinations based on the volumes that were mixed. Lowercase letters correspond to the same Tukey post-hoc test as those in Figure 2, so the absolute abundance can be directly compared between both plots (within one species).

S6: Consumer-Resource model

S6.1: Model Schematic

**Figure S6.1:** Schematic representation of the ordinary differential equation-based consumer-resource model. Solid arrows indicate uptake or production rate, arrow width scales qualitatively with magnitude (low, medium, large). Dashed lines represent sinks, dotted lines controls. NAG is taken up by the three species (PA = A, PX = B, SS = C in the equations) through concentration-dependent Monod kinetics at different maximum rates and half-saturation constants. Each species respires and dies at a fixed rate proportional to its biomass. Growth is limited through a biomass-feedback before all NAG is consumed, allowing the model to reach similar respiration levels in the 100% and 50% NAG treatments that we observed, while still having NAG left over (see S7). PA additionally produces hydrolytic enzymes, that move carbon from the chitin to the NAG pool, governed by Michaelis-Menten kinetics. Enzyme production is regulated by the concentration of NAG: PA allocates more C towards enzyme production if the concentration of NAG in the environment is low and vice-versa. Such mechanisms of NAG catabolites influencing chitinase synthesis negatively has been shown in several microbial systems [10–12]. This control on enzyme production by NAG concentration increases biomass and respiration when NAG is abundant, while it slows down biomass growth per unit of taken up NAG when NAG concentrations are low, as costly enzymes are being produced. Without this feedback, the chitin treatments would always reach the same cumulative respiration as the NAG treatments (although at a later timepoint outside of the simulation range), which is not what we observed. The model was implemented in R and simulated for all strain and substrate combinations for 350 hours with a stepsize of 0.001 using the ode() function of R package “deSolve” [13] with standard settings (lsoda integrator).

S6.2: Model Equations

**Figure S6.2:** Modelled carbon transformation in the biomass of species A (a). The input (NAG uptake, controlled by Monod saturation kinetics) is allocated towards biomass, maintenance respiration or enzyme production via the efficiency factor δ_A_. Mortality constantly reduces the biomass. We implemented a negative biomass feedback proportional to the total sum of biomass in the simulation to limit growth. This represents constraints beyond competition for substrate (NAG), such as for example spatial limitation. We also add a negative feedback on enzyme production where the enzyme production per unit of biomass is higher when there is less NAG in the environment. Substrate dynamics for the different scenarios are shown in b). When NAG is the only carbon source, all three species compete for it, and the one with the highest uptake rate (PX, species B in the model) outcompetes the others. If chitin is added (50% of total C), chitinases produced by PA (species A) are a public good, producing NAG from chitin from which all species benefit.

**Biomass:**

The change of biomass of enzyme producing species A (PA) is given by:

$\frac{dA}{dt}\text{= }\gamma_{1A}\text{ }\frac{NAG}{K_{A}\text{+}NAG}\text{ }A\text{- }\varepsilon\text{ }(A\text{+}B\text{+}C)\text{ }A\text{- }\sigma_{A}\text{ }A{\text{ - }\delta}_{A}\text{ }A\text{ }$(S1)

where the right-hand side terms of the equation correspond to: (1) Monod-like growth with the uptake rate dependent on NAG concentration; (2) to growth limitation feedback through the total biomass in a simulation (note that it includes all species present in a given scenario); (3) to mortality; (4) to metabolic expenses. Note that this term is related to cellular metabolism and consists mainly of enzyme production itself and respiration in general (maintenance and enzyme production, see Fig. S6.2). We assume this term is constant, independently from the amount of enzymes produced, representing a tradeoff in enzyme production and other cellular processes. As explained previously (S6.1), enzyme production is coupled to the concentration of NAG.

In a similar way we model the changes in biomass of the other species B (PX) and C (SS) as:

${\frac{dB}{dt}\text{=}\text{ }\gamma}_{B}\text{ }\frac{NAG}{K_{B}\text{+}NAG}\text{ }B\text{- }\varepsilon\text{ }\left( A\text{+}B\text{+}C \right)\text{ }B\text{- }\sigma_{B}\text{ }B{\text{ - }\delta}_{B}\text{ }B\text{ }$ (S2)

${\frac{dC}{dt}\text{=}\text{ }\gamma}_{C}\text{ }\frac{NAG}{K_{C}\text{+}NAG}\text{ }C\text{- }\varepsilon\text{ }\left( A\text{+}B\text{+}C \right)\text{ }C\text{- }\sigma_{C}\text{ }C{\text{ - }\delta}_{C}\text{ }C\text{ }$ (S3)

Note that in our formulation, all species are constrained in the same way by the total biomass in the system, sharing a common parameter ε. In monoculture scenarios, this constraint reduces to self-limitation by the species own biomass only.

**Substrates:**

Substrate degradation is modeled according to Michaelis-Menten kinetics, where hydrolytic enzymes decompose the chitin pool at a fixed specificity and speed:

$$\frac{dChi}{dt}\text{ = - }\frac{\alpha\text{ }E\text{ }Chi}{\kappa\text{ + }Chi}\text{ }(S4)$$

This carbon is moved to the NAG pool, from which it is taken up by the species at their individual uptake rate:

$$\frac{dNAG}{dt}\text{ = }\frac{\alpha\text{ }E\text{ }Chi}{\kappa\text{ + }Chi}\text{ - }\gamma_{A}\text{ }\frac{NAG}{K_{A}\text{+}NAG}\text{ }A\text{ - }\gamma_{B}\text{ }\frac{NAG\text{ }}{K_{B}\text{+}NAG}B\text{ - }\gamma_{C}\text{ }\frac{NAG\text{ }}{K_{C}\text{+}NAG}C\text{ }(S5)\text{ }$$

Where γ_i_ represents the maximum uptake rate of species *i* (A, B or C), K*_i_* represents the half saturation (substrate/NAG concentration at which this uptake rate is γ_i_/2). This results in a linear uptake regime at low NAG concentrations and a saturating regime at high substrate concentrations, as described by Monod kinetics, see Fig. S6.4.

**Enyzmes:**

Hydrolytic Enzymes are produced by species A (see above) and decay at a fixed rate ρ:

$$\frac{dE}{dt}\text{ = }\frac{\theta}{1\text{+ }\left( NAG\text{ }\kappa_{eA} \right)}\text{ }\delta_{A}\text{ }A\text{ - }\rho\text{ }E\text{ }(S6)$$

**Sinks:**

Respiration is tracked as the sum of all respiration terms:

$$\frac{dR}{dt}\text{ = }\left( 1\text{- }\frac{\theta}{1\text{+}\left( NAG\text{ }\kappa_{eA} \right)} \right)\delta_{A}\text{ }A\text{ + }\delta_{B}\text{ }B\text{ + }\delta_{C}\text{ }C\text{ }(S7)$$

Necromass is finally tracked as the sum of all mortality terms:

$$\frac{dNec}{dt}\text{ = }\sigma_{A}\text{ }A\text{ + }\sigma_{B}\text{ }B\text{ + }\sigma_{C}\text{ }C\text{ }(S8)$$

S6.3: Initial Conditions

| Substrate Condition | NAG | Chi | Residual NAG [% of total C] |
| --- | --- | --- | --- |
| NAG | 6677000 | 0 | - |
| NAG+Chitin | 3341171 | 3335829 | 0.08 |
| Chitin | 5341.6 | 6671658.4 |  |

Initial conditions were estimated from the initial total C amounts in the experiment, calculated in ng C. For this, a carbon-content of 43.93% of NAG was assumed, which was determined by measuring the pure N-acetylglucosamine used in this study on an elemental analyzer (EA 1110, CE Instruments). For convenience, everything was scaled to 1 ml (from 500 µl volume of each replicate in the experiment). To compare the simulated respiration with the measured, it was then simply halved again. For the biomass, we assumed a C content of 12.4 fg C * cell^-1^ [14], which resulted in roughly 1 ng C total biomass with our measured initial absolute abundances. For the biodiversity treatments, this total initial biomass was split evenly amongst the functional groups (0.5/0.5 for the duos, 0.33/0.33/0.33 for the trios). We assumed that the chitin we used was not completely free of residual NAG [15] and estimated the amount of residual NAG based on the measured respiration of the non-chitinase producing strain PX with chitin as its sole C source (because we assume all C comes from that residual NAG in that case). This residual NAG is also added in the NAG+chitin scenario, scaled with the total amount of chitin (which is half of that in the Chitin treatment to keep total C constant).

S6.4: Functional Groups

| Strain | Hydrolytic Enzymes | Growth on NAG |
| --- | --- | --- |
| PA (A) | + | + |
| PX (B) | - | ++ |
| SS (C) | - | (+) |

Qualitative assignment of functional groups to the different strains used in the experiment (++ = high, + = medium, (+) = low, - = none). This was derived from different measurements after the experiments and did not influence prior strain selection: Only PA produced hydrolytic chitinases in the chitinase assay (Figure 3). PX utilizes NAG the best, followed by PA, while SS barely respires and grows in mono-culture on NAG (Figure 1).

S6.5: Parameters and their Values

| Parameter | Description | Unit |  | Value |
| --- | --- | --- | --- | --- |
| α | V_max_ Chitinase | ng C * h^-1^ * ml^-1^ | optimized | 495890.2344 |
| κ | K_m_ Chitinase | ng C * ml^-1^ | fixed | 1880204 |
| ρ | Decay Chitinase | h^-1^ | optimized | 0.0060 |
| γ_A_ | Maximum NAG uptake rate by A,B,C | h^-1^ | optimized | 0.5375 |
| γ_B_ |  |  | optimized | 0.7677 |
| γ_C_ |  |  | optimized | 0.1885 |
| κ_A_ | Half-saturation constant NAG uptake for A,B,C | ng C * ml^-1^ | optimized | 145.0574 |
| κ_B_ |  |  | optimized | 8582.6622 |
| κ_C_ |  |  | optimized | 10000 |
| κ_eA_ | NAG control on hydr. Enzyme production by A | ng C^-1^ * ml^-1^ | fixed | 25 |
| θ | Proportion of biomass of Strain A allocated to hydr. enzyme production | - | optimized | 0.00047 |
| δ_A_ | Efficiency factor of Strain A,B,C | h^-1^ | optimized | 0.0049 |
| δ_B_ |  |  | optimized | 0.0130 |
| δ_C_ |  |  | optimized | 0.0016 |
| ε_A_ | Scaling factor negative feedback biomass | ng C^-1^ * h^-1^ * ml^-1^ | optimized | 4.856 x 10^-5^ |
| ε_B_ |  |  |  |  |
| ε_C_ |  |  |  |  |
| σ_A_ | Death rate of Strain A,B,C | h^-1^ | optimized | 0.0739 |
| σ_B_ |  |  | optimized | 0.0146 |
| σ_C_ |  |  | optimized | 0.0148 |

Final parameter sets for each scenario, values for optimized parameters are the maximum a posteriori estimation (MAP). As respiration was the only time-resolved measurement in this experiment, it was used to calibrate unfixed parameters in the mono-cultures. For this, a Markov-Chain Monte-Carlo approach was applied, maximizing the log-likelihood of the simulated respiration data given the experimental respiration mean and standard deviation with a certain set of parameter values (see S8.6 for detailed description of the approach). We also carried out a preliminary manual exploration of the parameter space, complemented by measurements from other experiments and literature research to include plausible bounds for the parameter values. For the uptake rate, we chose an upper value of ~0.77, corresponding to a maximum doubling time of 0.9 * h^-1^, which we deemed a reasonable value for a fast growing bacterium in near-optimal substrate conditions [16]. For the uptake half-saturation constants, we assumed a parameter range around ~10^3^ ng C * ml^-1^, which corresponds to a concentration of around 10 µM [17]. This value is published for glucose, however as it is structurally similar to NAG we assume similar uptake systems with similar properties.

As it had the largest effect on remaining NAG in the simulations, prior limits for the negative feedback ε were chosen in a range that ensured similar consumption of NAG compared to the experiment. This was estimated by calculating the proportion of mean consumed NAG by PX in the final NAG and NAG+chitin supernatants relative to the respective uninoculated controls (see S7) and applying this proportion to the initial NAG in the model. The chitinase half saturation constant K_m_ was approximated from the literature for hydrolytic chitinases [18].

Using these constraints, we optimized the uptake rate γ, efficiency δ, negative feedback ε and death rate σ for PX to match the respiration in the NAG-only treatment. The amount of residual NAG in chitin was then adjusted manually to match the total amount of measured respiration of PX in the chitin-only treatment. As we assume maximum possible uptake rates in NAG and NAG+chitin due to high NAG concentration, we optimized the half-saturation κ_B_ (and κ_A_ further below) in these chitin conditions to best match the measured respiration curve shape as it only has an effect on uptake rates in low-NAG conditions (S6.4).

We then set the biomass-scaling feedback parameters ε_A_ and ε_C_ to the same value as what the algorithm found for PX, assuming that the growth limitation was based on total biomass in a treatment and the same for all three species. Next, we optimized the NAG uptake γ, efficiency δ and death rate σ for PA and SS (species A and C) in NAG-only conditions. Finally, enzyme production θ, V_max_, enzyme decay ρ and uptake half saturation κ_A_ were optimized for the PA monoculture growing on chitin only, keeping all previously (in NAG-only) calibrated parameters at their optimal value. Note that the parameter that controls the NAG-feedback on enzyme production κ_eA_ was set to an arbitrary number that obtained saturation of respiration on lower levels in chitin than in NAG. For S. soli, we set κ_C_ to a value an order of magnitude above the other two species before parametrization in NAG, as the approach we used for PA did not work here due to minimal respiration of SS in both NAG and chitin.

S6.6: Uptake kinetics

**Figure S6.6:** Uptake rate relative to NAG concentration for PA and PX, based on the parameters obtained via MCMC. PX thrived when NAG was abundant, while the high respiration of PA on chitin suggests an advantage of the species under such conditions. This led us to parametrize different uptake dynamics for each species, which are driven by the maximum uptake rate γ_A,B_ and the uptake half-saturation constant κ_A,B_. The figure illustrates the realized uptake rate in relation to NAG concentration for both species, given their half-saturation constants - where PX seems to thrive in high nutrient conditions while PA already grows well when low amounts of NAG are present (consistent with its degrader role). This results in PX outcompeting PA in high-NAG conditions in the model and PA outcompeting PX in low-NAG conditions (see also S6.7), with resulting predicted respiration mirroring the mono-cultures of the respective dominating species. Interactions that go beyond this dynamic competition for NAG change the measured respiration compared to this simulation. Note that for S. soli, κ_C_ was set to a high value, which together with a lower γ_C_ results in much lower uptake, growth and respiration on NAG, just as we observed.

S6.7: Simulated biomass dynamics

**Figure S6.7:** Simulated biomass for the first 50 hours for species combinations (excluding mono-cultures for better visibility) across all substrates. Model settings for these simulations are the same as those used for Fig. 5. This shows how the dominating species for the respective substrate condition (see S6.6) outcompetes the others quickly and does not allow them to establish beyond a minimal peak in the first 20 to 30 hours.

S7: Final NAG concentration in the supernatant

**Figure S7:** Final N-acetylglucosamine (NAG) concentrations in the supernatants at the incubation endpoint for all species and substrate combinations, obtained via PMP-derivatization and UPLC-Orbitrap/MS (see S8.3 for details). The dashed line indicates the measured value in the sterile controls. Interpretation of this data is obscured by multiple factors: Values higher than controls are likely due to evaporation from the microwells during the course of the incubation, which increased concentrations over time. This is likely not constant across one plate, but differs from the edges to the inside, and positions of treatments varied across plates. N-acetylglucosamine is also a part of peptidoglycan, which forms the cell wall of bacteria. NAG is therefore constantly consumed, produced and recycled, obscuring potential treatment effects. The data however shows that NAG was still present at the incubation endpoint in most cases.

S8: Supplementary Methods

S8.1: Cumulative Respiration of consortia on different substrates

Strains were obtained from the German Collection of Microorganisms and Cell Cultures GmbH (DSMZ) and maintained in their respective optimal medium (DSMZ M1 for PA, LB for PX and R2A for SS) at 28°C prior to the experiment. The bacteria were pre-grown in triplicates (named A,B and C) in optimal medium until exponential phase (1 day for PA and PX, 3 days for SS). SS and PA were previously maintained in liquid culture, PX was started from a cryo-stock as this yielded the most reliable growth results. Cultures were then harvested by centrifuging (8000 *x g* for 10 minutes) and re-suspending the pellet in 30 ml of carbon-free M9 minimal medium [19] and left at room temperature for two hours for remaining nutrients to be metabolized. After a 1:10 serial dilution, cells were then counted in one replicate of each strain that had an OD_600_ of about 0.1 using a Quantom Tx cell counter (Logos Biosystems) according to the manufacturer’s protocol to ensure cell concentrations in the same order of magnitude between the cultures. The chosen dilutions were then mixed in the same ratio in all possible combinations. To reduce the total amount of combinations, we only mixed one replicate of one culture with one replicate of another culture, not with all replicates of that second culture (for example PA-A was only mixed with PX-A and not with PX-B or PX-C).

Colloidal chitin (CC) was prepared from shrimp shells [20]. In brief, chitin from shrimp shells (Sigma Aldrich) was stirred with 12M HCl for 1 hour, filtered through cheese cloth and then mixed with 2l MQ and left to settle overnight at 4°C. Then, it was again filtered, washed with MQ until the pH was neutral and autoclaved. MicroResp^tm^ indicator plates were prepared and stored according to the manufacturer’s instruction. 96-deepwell plates (Greiner) were filled with four different substrates (M9, M9 + NAG (2% w/v), M9 + CC (2% w/v), M9 + CC + NAG (1% + 1% w/v), 380 µl per well). After UV-irradiating the plates in a laminar flow hood for 30 minutes, 120 µl of all biodiversity treatments were added in triplicates for each substrate. The indicator plate absorbance baseline at 540 nm was measured in a plate reader (Tecan SPARK) and MicroResp setups were assembled and incubated for two weeks at 28°C, shaking at 100 rpm. Every 24 hours, the indicator plate was removed, measured at 540 nm, and replaced with a fresh plate to avoid saturation of the indicator and allowing air exchange. At the end of the incubation, the contents of the wells were transferred into reaction tubes and frozen at -20°C. Technical replicates were pooled in this step to obtain enough sample volume for subsequent measurements. These pooled samples were later separated into a pellet containing the bacterial cells plus any remaining solid substrate and the supernatant containing dissolved metabolites and degradation products by centrifugation (8000 *x g* for 10 minutes).

The absorbance change of the indicator plates was corrected by the background colour change in non-inoculated blanks for each substrate and converted to %CO_2_ in the headspace according to the MicroResp user manual. This was then converted to µg CO_2_ for each incubation time interval using the following formula derived from an earlier calibration:

$$\mu g\text{ }{CO}_{2}C\text{ = }\frac{{\text{\%}CO}_{2}}{100}\text{ }\times\text{ }945\text{ }\times\frac{44}{22.4}\text{ }\times\frac{12}{44}\text{ }\times\frac{273}{273\text{+}28}\text{ }$$

The calculated CO_2_ production was then accumulated over the whole incubation period. This value was corrected for each timepoint and technical replicate by the mean background respiration of the strains in the respective combinations in M9 minimal media without any added carbon to obtain the substrate-induced respiration response of the bacteria.

Nonlinear least-squares regressions were fitted to the cumulative respiration for each strain/substrate combination using the nls() function of the package “stats” on biological replicates. Different curve shapes were fitted with self-starting models (SSlogis() for a logistic model, SSasymp() for an asymptotal model, SSgompertz() for a Gompertz-type growth curve model and SSexpf() for an exponential model) and the best model based on lowest deviance from the data (checked with function deviance()) was chosen. Autocorrelation over time was assessed with function “acf” of R package “stats”. These were then compared to a null model that was fit to all data of each biodiversity treatment/substrate combination (e.g. Mono/Chitin) using a F-test (function anova() of R package “stats”) to check for significant effects of the strain combinations on respiration (α = 0.05). P-values were adjusted for multiple testing with Benjamini-Hochberg correction.

S8.2: Final Community Composition

DNA was extracted from 50 µl of the pellets resuspended in 1 ml of DNAse-free water (Carl Roth) by heating to 95°C in a thermal cycler (BioRad Laboratories T100) for 10 minutes. The DNA content was then quantified using the Qubit high-sensitivity assay (Thermo Fisher Scientific) prior to ddPCR. Samples with a concentration higher than 0.05 ng * µl^-1^ were diluted to that concentration to avoid droplet overloading, samples with lower concentration were used undiluted. Digital droplet PCR was then performed on the diluted DNA extracts similar to Martin et al. [21] with primers 338F/805R using a QX200 droplet generator and droplet reader (BioRad). The total volume of each reaction was 22 µl, consisting of 1x QX200 ddPCR EvaGreen Supermix (BioRad), 0.1 µmol*l^-1^ forward and reverse primer respectively and 2 µl DNA template. For samples with low DNA concentrations, 4 µl template were used, but total volume was kept at 22 µl. Droplets were subjected to PCR using the same conditions as previously described [21]. After the PCR, the droplets were left at 4°C overnight before reading them. Fluorescence thresholds deciding whether a droplet was positive or negative were assigned consistently across samples using the histograms provided by the QuantaSoft software (BioRad, version 1.0.596). Copy numbers per µl template were calculated according to Angel & Petrova [22] and corrected for previous dilution. Extracts were in addition sent to the Joint Microbiome Facility (JMF, Vienna, Austria) for 16S rRNA amplicon sequencing [23]. Microplate handling introduced some contaminations, likely caused by condensation and the frequent absorbance plate exchange. Cross contaminated samples were removed from further analysis. We assumed remaining rare contaminant amplicon sequencing variants (ASVs) to be artifacts or introduced to the samples after the experiment that did not affect the generated data, as their abundance was much lower than that of our targets. Contaminant ASVs with more than 1% relative abundance made up a minor contribution to total biomass (Fig. 2) and samples where our target strains were outcompeted were removed from analysis. ASVs that made up a higher abundance and were assigned the same genus as our target strains but lacked a species-level assignment were compared to the reference genome using the basic local alignment search tool (BLAST) of the NCBI to confirm their similarity of the 16S sequence was higher than 99% to the target sequence.

S8.3: Chitin decomposition products

Chitobiose and chitotriose concentrations were measured using a UPLC-Orbitrap/MS method combined with pre-column 1-phenyl-3-methyl-5-pyrazolone (PMP)-derivatisation described in Salas et al. [24]. Briefly, 100 µl of the supernatant of pooled technical replicates was subjected to 100 µl 0.5 M PMP and 200 µl NH_3_ and heated to 70°C for 20 minutes. After reaching room temperature, 200 µl of formic acid were added, and excess PMP was removed by extracting the samples twice with chloroform and filtering them through a 0.2 µm cellulose-acetate syringe filter (LLG GmbH). The samples were then measured on a UPLC Ultimate 3000 system attached to an Orbitrap Q Exactive HRMS system (both Thermo Fisher Scientific) using an electrospray-ionization source. Used chromatography and mass spectrometry settings were reported previously [24]. N-acetylglucosamine, Chitobiose and Chitotriose were quantified at masses 552.2452, 755.3249 and 958.4038 respectively using FreeStyle version 1.7 (Thermo Fisher Scientific). Pure N-acetylglucosamine (Sigma), N,N,N-triacetylchitotriose and N,N-diacetylchitotriose (both Biosynth, UK) were used as standards for substance quantification.

S8.4: Chitinase activity

Cultures were grown and mixed together in the same way as in the MicroResp experiment. 190 µl of substrate (M9 + 2% NAG, M9 + 1% NAG + 1% CC, M9 + 2% CC) were inoculated with 60 µl of culture mix each in microtiter plates. The plates were then incubated on a shaker (100 rpm) at 28°C. At day 10, three different replicates of each treatment were transferred into reaction tubes, centrifuged (12000 rpm for 5 minutes) and extracellular chitinase activity was assessed with 4-methylumbelliferone (4-MUF)-labelled fluorogenic substrates [25]. In brief, 10 µl of sample supernatant were added to a sodium acetate buffer (50 mM, pH = 5) containing either 4-MUF-labelled N-acetylglucosamine, chitobiose or chitotriose (0.2 mg * ml^-1^ each, Sigma-Aldrich). Enzymatic activity was then assessed by measuring fluorescence over time, which increases when 4-MUF is released by enzymatic action. MUF release was quantified by calibrating with the fluorescence of a pure MUF standard series. Fluorescence was measured at 365/450 nm (excitation/emission) in a plate reader in black microtiter plates (Greiner).

S8.5 Cell-free spent media experiment

The species were pre-grown in liquid culture in 50 ml of the same media as in the MicroResp experiment (M9 + 2% NAG, M9 + 1% chitin + 1% NAG, M9 + 2% chitin) shaking at 100 rpm and 28°C for 10 days. For each medium, an uninoculated control was added, which served as fresh, unspent medium later in the experiment. The cultures were then centrifuged (8000 g for 10 minutes) and the supernatant was filtered through sterile 0.22 µm cellulose-acetate syringe filters (Chromafil, Macherey-Nagel). The obtained cell-free supernatants were stored at 4°C until further use. Note that any present chitin particles (leftover or in the uninoculated controls) were filtered out as well. Fresh cultures (3 biological replicates) were started in optimal medium from three different glycerol stocks each and allowed to reach exponential phase before harvesting them. Cultures were centrifuged, washed the same way as in the MicroResp experiment. Cell numbers were again checked and dilutions with similar abundances were chosen as inoculate. Microtiter plates containing wells filled with 190 µl of cell-free spent media of each substrate type and donor species (including uninoculated controls) were then inoculated with 60 µl of harvested, washed and diluted culture in triplicates. These were sealed with parafilm and incubated on a shaker at 28°C for 10 days. OD_600_ was recorded every 24h using a plate reader (Tecan SPARK). Prior to each measurement, the lid of the microtiter plate was replaced in a laminar flow hood to remove built-up condensation which otherwise interfered with the absorbance readings. Optical density was averaged over technical replicates for each timepoint, and the maximum value across the whole timeseries was extracted and plotted in Figure 4.

S8.6: Model Parameter Optimization

Parameter values of the consumer resource model were optimized by applying a Differential Evolution Markov-Chain Monte Carlo approach implemented in R. For each parametrization step (see S6.5), some parameters were fixed and others varied using the runMCMC() function of package BayesianTools [26]. Rough lower and upper values were estimated by manual simulations and MCMC trials first and used as initial priors for the algorithm to reduce runtime and improve convergence (see S6.5). The algorithm calculates a set of parameters based on the priors and previous iterations and then simulates the model with these parameters. Then, the log likelihood of the simulated respiration given the measured respiration (assuming a normal distribution) was calculated and maximized. The MCMC algorithm was run with 3 chains and 100000 iterations each, which was enough for it to converge to an optimal mean parameter value (with the highest log likelihood) in testing while still maintaining acceptable computation time on a standard office PC (see Fig. S8.6 for an example of the resulting parameter distribution). The maximum a posteriori (MAP) value of the distribution was then used as the final parameter value, obtained with function MAP().

**Figure S8.6:** Chain traces and parameter distribution for an exemplary parametrization run using the MCMC approach, optimizing two parameters at once.

S8.7: Statistical analysis

All plotting and statistical analysis were done in R (4.1.2) using RStudio (2023.06.0). NLS models were checked for autocorrellation by calculating the average value across the entire timeseries using function “acf” of the “stats” package. Before performing an ANOVA, data was checked for homogeneity of variance using Levene’s test. Normality of residuals was assessed by visual inspection of the quantile-quantile plot and a Shapiro-Wilk test. If necessary, data was log-transformed to meet ANOVA assumptions. Tukey’s honest significant difference test was afterwards performed to assess significant differences between groups. If requirements were not met, a nonparametric Kruskal-Wallis test followed by Dunn’s post-hoc test was instead performed. Effect size η^2^ was calculated with function eta_squared() for ANOVA or function kruskal_effsize() for the Kruskal-Wallis tests, both from package “rstatix” [27]. Effects or differences between groups were deemed significant below a p-value threshold of 0.05.

S8.8 Definition of interactions for Fig. 6

Biomass effects of one species on another were assessed by evaluating the biomass change in the duo-culture to the respective monoculture: higher biomass indicated a promoting and a lower a suppressing effect. Biomass differences below 10% were considered negligible with no interaction assigned in this case (e.g., PX in PX+SS-NAG, -3.7% compared to monoculture - Fig. 2b and PA in PA+SS-chitin - Fig. 2h, -3.5%). For *S. soli*, endpoint detectability was considered positive due to its frequent extinction. Interactions affecting respiration were defined by maximum values compared to monoculture, however only if the species was detectable at the end, e.g. we assigned a positive effect of respiration on PA in the PA+SS NAG combination (Fig. 5e), but not on SS. Additionally, earlier onset of respiration in PA+SS-Chitin compared to PA in monoculture (see Fig. 1 c,f or Fig. 5 s,o) was also deemed a positive effect. Here, we employed no threshold as for biomass as differences were less narrow. For growth on waste metabolites, effects were evaluated based on the difference to each species grown in its own waste, again without a threshold. We excluded the NAG+chitin treatment here, because NAG depletion confounded interaction effects.

References

1.	Beier S, Bertilsson S. Bacterial chitin degradation-mechanisms and ecophysiological strategies. *Frontiers in Microbiology* 2013;**4**:149. https://doi.org/10.3389/fmicb.2013.00149.

2.	Guseva K, Mohrlok M, Alteio L, Schmidt H, Pollak S, Kaiser C. Bacteria face trade-offs in the decomposition of complex biopolymers. *PLoS Comput Biol* 2024;**20**(8):e1012320. https://doi.org/10.1371/journal.pcbi.1012320.

3.	Vaaje-Kolstad G, Westereng B, Horn SJ, Liu Z, Zhai H, Sørlie M *et al.* An Oxidative Enzyme Boosting the Enzymatic Conversion of Recalcitrant Polysaccharides. *Science* 2010;**330**(6001):219–22. https://doi.org/10.1126/science.1192231.

4.	Jiang WX. A pathway for chitin oxidation in marine bacteria. *Nature Communications* 2022;**13**:5899.

5.	Caspi R, Billington R, Keseler IM, Kothari A, Krummenacker M, Midford PE *et al.* The MetaCyc database of metabolic pathways and enzymes - a 2019 update. *Nucleic Acids Research* 2020;**48**(D1):D445–53. https://doi.org/10.1093/nar/gkz862.

6.	Karp PD, Midford PE, Billington R, Kothari A, Krummenacker M, Latendresse M *et al.* Pathway Tools version 23.0 update: software for pathway/genome informatics and systems biology. *Briefings in Bioinformatics* 2021;**22**(1):109–26. https://doi.org/10.1093/bib/bbz104.

7.	Roman DL, Ostafe V, Isvoran A. Deeper inside the specificity of lysozyme when degrading chitosan. A structural bioinformatics study. *Journal of Molecular Graphics and Modelling* 2020;**100**:107676. https://doi.org/10.1016/j.jmgm.2020.107676.

8.	Zhang H, Yohe T, Huang L, Entwistle S, Wu P, Yang Z *et al.* dbCAN2: a meta server for automated carbohydrate-active enzyme annotation. *Nucleic Acids Research* 2018;**46**(W1):W95–101. https://doi.org/10.1093/nar/gky418.

9.	Barrett K, Lange L. Peptide-based functional annotation of carbohydrate-active enzymes by conserved unique peptide patterns (CUPP). *Biotechnol Biofuels* 2019;**12**(1):102. https://doi.org/10.1186/s13068-019-1436-5.

10.	Bakare MK, Adewale IO, Ajayi AO, Okoh AI, Shonukan OO. Regulatory mutations affecting the synthesis of cellulase in Pseudomonas fluorescens. *African Journal of Biotechnology* 2005;**4**(8):838–43.

11.	Green VE, Klancher CA, Yamamoto S, Dalia AB. The molecular mechanism for carbon catabolite repression of the chitin response in Vibrio cholerae. *PLoS Genet* 2023;**19**(5):e1010767. https://doi.org/10.1371/journal.pgen.1010767.

12.	Stewart BJ, Leatherwood JM. Derepressed synthesis of cellulase by Cellulomonas. *J Bacteriol* 1976;**128**(2):609–15. https://doi.org/10.1128/jb.128.2.609-615.1976.

13.	Soetaert K, Petzoldt T, Setzer RW. Solving Differential Equations in *R* : Package **deSolve**. *J Stat Soft* 2010;**33**(9). https://doi.org/10.18637/jss.v033.i09.

14.	Fukuda R, Ogawa H, Nagata T, Koike I. Direct Determination of Carbon and Nitrogen Contents of Natural Bacterial Assemblages in Marine Environments. *Applied and Environmental Microbiology* 1998;**64**(9):3352–8. https://doi.org/10.2331/suisan.77.134.

15.	Pontrelli S, Szabo R, Pollak S, Schwartzman J, Ledezma-Tejeida D, Cordero OX *et al.* Metabolic crossfeeding structures the assembly of polysaccharide degrading communities. *Science Advances* 2022;**8**:eabk3076. https://doi.org/10.1126/sciadv.abk3076.

16.	Weissman JL, Hou S, Fuhrman JA. Estimating maximal microbial growth rates from cultures, metagenomes, and single cells via codon usage patterns. *Proc Natl Acad Sci USA* 2021;**118**(12):e2016810118. https://doi.org/10.1073/pnas.2016810118.

17.	Szenk M, Dill KA, De Graff AMR. Why Do Fast-Growing Bacteria Enter Overflow Metabolism? Testing the Membrane Real Estate Hypothesis. *Cell Systems* 2017;**5**(2):95–104. https://doi.org/10.1016/j.cels.2017.06.005.

18.	Du J, Duan S, Miao J, Zhai M, Cao Y. Purification and characterization of chitinase from *Paenibacillus* sp. *Biotech and App Biochem* 2021;**68**(1):30–40. https://doi.org/10.1002/bab.1889.

19.	Souza CP, Burbano-Rosero EM, Almeida BC, Martins GG, Albertini LS, Rivera ING. Culture medium for isolating chitinolytic bacteria from seawater and plankton. *World Journal of Microbiology and Biotechnology* 2009;**25**(11):2079–82. https://doi.org/10.1007/s11274-009-0098-z.

20.	Murthy N, Bleakley B. Simplified Method of Preparing Colloidal Chitin Used For Screening of Chitinase- Producing Microorganisms. *The Internet Journal of Microbiology* 2012;**10**(2):1–5.

21.	Martin V, Schmidt H, Canarini A, Koranda M, Hausmann B, Müller CW *et al.* Soil cover shapes organic matter pools and microbial communities in soils of maritime Antarctica. *Geoderma* 2024;**446**:116894. https://doi.org/10.1016/j.geoderma.2024.116894.

22.	Angel R, Petrova E. Quantification of 16S rRNA Gene Copies Using ddPCR (EvaGreen-based assay: 338F-805R) v3. *ProtocolsIo* 23 Nov. 2020:1–13. https://doi.org/10.17504/protocols.io.bpy6mpze.

23.	Pjevac P, Hausmann B, Schwarz J, Kohl G, Herbold CW, Loy A *et al.* An Economical and Flexible Dual Barcoding, Two-Step PCR Approach for Highly Multiplexed Amplicon Sequencing. *Frontiers in Microbiology* 2021;**12**:669776. https://doi.org/10.3389/fmicb.2021.669776.

24.	Salas E, Gorfer M, Bandian D, Wang B, Kaiser C, Wanek W. A rapid and sensitive assay to quantify amino sugars, neutral sugars and uronic acid necromass biomarkers using pre-column derivatization, ultra-high-performance liquid chromatography and high-resolution mass spectrometry. *Soil Biology and Biochemistry* 2023;**177**:108927. https://doi.org/10.1016/j.soilbio.2022.108927.

25.	Marx MC, Wood M, Jarvis SC. A microplate fluorimetric assay for the study of enzyme diversity in soils. *Soil Biology and Biochemistry* 2001;**33**(12–13):1633–40. https://doi.org/10.1016/S0038-0717(01)00079-7.

26.	Hartig F, Minunno F, Paul S. *BayesianTools: General-Purpose MCMC and SMC Samplers and Tools for Bayesian Statistics*, version 0.1.4. n.d.

27.	Kassambara A. *Rstatix: Pipe-Friendly Framework for Basic Statistical Tests*. 2023. https://rpkgs.datanovia.com/rstatix/.
